# Acute-Phase Dengue Antibody Profiles in Pediatric Patients: Influence on Viremia and Disease Manifestations

**DOI:** 10.3390/v18070741

**Published:** 2026-07-03

**Authors:** Florencia A. Bonnin, Agostina Bruno, María Manuela Bono, Carolina A. Lucero, Ludmila Niño, Mariela Del Giudice, Diego E. Álvarez, Eduardo L. López, Cybele C. García, Marcelo O. Quipildor, Laura B. Talarico

**Affiliations:** 1 Laboratorio de Investigaciones Infectológicas y Biología Molecular (LIIBM), Consejo Nacional de Investigaciones Científicas y Técnicas (CONICET), Hospital de Niños Dr. Ricardo Gutiérrez, Ciudad Autónoma de Buenos Aires 1425, Argentina; florenciabonn616@gmail.com (F.A.B.); elopez2676@gmail.com (E.L.L.); 2 Departamento de Química Biológica, Facultad de Ciencias Exactas y Naturales, Universidad de Buenos Aires (UBA), Ciudad Autónoma de Buenos Aires 1428, Argentina; 3 Hospital San Vicente de Paul, San Ramón de la Nueva Orán 4530, Salta, Argentina; bagostina@hotmail.com (A.B.); mariamanuelabono@outlook.com.ar (M.M.B.); 4 Instituto de Investigaciones Biotecnológicas (IIBIO), Universidad Nacional de San Martín (UNSAM)-CONICET, San Martín 1650, Buenos Aires, Argentina; clucero@iib.unsam.edu.ar (C.A.L.); ludmilaninio2@gmail.com (L.N.); mdelgiudice@iib.unsam.edu.ar (M.D.G.); dalvarez@iibintech.com.ar (D.E.Á.); 5 Departamento de Medicina, Programa de Enfermedades Infecciosas, Hospital de Niños Ricardo Gutiérrez, Universidad de Buenos Aires, Ciudad Autónoma de Buenos Aires 1425, Argentina; 6 Instituto de Química Biológica de la Facultad de Ciencias Exactas y Naturales (IQUIBICEN), UBA-CONICET, Ciudad Autónoma de Buenos Aires 1428, Argentina; cygarcia@qb.fcen.uba.ar

**Keywords:** dengue virus, immune status, neutralizing antibody, pediatric, viremia, clinical disease

## Abstract

Secondary dengue infections are often linked to more severe clinical outcomes, yet pre-existing antibodies may exert either protective or pathogenic effects. To evaluate the role of acute-phase dengue antibodies in pediatric dengue, we analyzed clinical and laboratory features, viremia, and antibody profiles in children infected with DENV-1. We conducted a retrospective study of patients under 18 years diagnosed with DENV-1 in Salta, Argentina. Viremia was quantified by real-time RT-PCR; acute-phase IgG antibodies (within 7 days from symptom onset) against DENV, DENV-1, and DENV-NS1 were measured by immunoassays, and neutralizing antibodies by plaque reduction neutralization tests. Among 151 patients (median age 12 years), 62% presented dengue with warning signs and one case progressed to severe dengue. Viremia was higher in probable primary infections than in probable secondary infections and did not correlate with disease severity. Probable secondary infections were characterized by acute-phase antibody profiles that did not associate with DENV viremia. Age-stratified analyses revealed that adolescents exhibited higher viremia levels than younger children in probable primary infections, while viremia levels were comparable across age groups in probable secondary infections. Furthermore, children younger than 10 years displayed acute-phase antibody levels similar to those of adolescents. In adolescents with probable secondary infections, anti-DENV and anti-DENV-1 IgG were inversely correlated with platelet counts, whereas neutralizing and anti-DENV-NS1 antibodies showed no association. Collectively, these findings indicate that probable secondary DENV infections in our pediatric cohort were characterized by acute-phase antibodies that were not associated with viremia control, and that in adolescents, anti-DENV and anti-DENV-1 IgG antibodies likely associated with platelet depletion. These results highlight important implications for vaccine design, underscoring the need for vaccines that elicit strong neutralizing responses while minimizing cross-reactivity and the risk of antibody dependent enhancement.

## 1. Introduction

Dengue virus (DENV) infection is one of the most prevalent arthropod-borne viral diseases worldwide and represents a major, expanding public health challenge. It is estimated to cause approximately 390 million infections annually, with children and adolescents bearing a substantial proportion of the global burden [1,2]. Dengue imposes considerable health and economic costs, accounting for millions of hospitalizations each year and contributing significantly to disability-adjusted life years in endemic regions [3,4]. DENV transmission is now established in more than 100 countries, with the highest burden reported in the Americas, Asia, and the Western Pacific. The co-circulation of four antigenically distinct serotypes (DENV-1 to DENV-4) drives recurrent epidemics and increases the risk of severe disease [5]. In 2024, over 14 million cases and more than 11,000 deaths were officially reported worldwide, marking the highest incidence ever documented [6]. The continued spread of dengue is facilitated by the expanding geographic distribution of *Aedes aegypti* and *Aedes albopictus*, the principal vectors of DENV, a process accelerated by climate change, rapid urbanization, and globalization [7]. 

Human infection with DENV produces a wide clinical spectrum, ranging from mild febrile illness with rash and musculoskeletal pain to dengue with warning signs (DWWS), which may include hemorrhage, edema, hepatomegaly, and thrombocytopenia, and severe disease characterized by shock, organ failure, and life-threatening bleeding [8]. Primary infection confers lifelong immunity only to the infecting serotype, while sequential heterotypic infections have been associated with severe disease through antibody-dependent enhancement (ADE) [9]. In this process, non-neutralizing heterologous antibodies facilitate viral entry into Fcγ receptor-bearing cells, amplifying replication and triggering pro-inflammatory cytokine and complement activation, which compromise vascular integrity and lead to plasma leakage, a hallmark of severe dengue [10]. In contrast, neutralizing antibodies can provide protective immunity against secondary infection, although their effectiveness varies by serotype and prior exposure history [11,12]. The balance between protective neutralization and pathogenic ADE underscores the complexity of dengue immunology and the challenges for vaccine development.

Given dengue’s high morbidity, potential for severe outcomes, and substantial impact on healthcare systems, the disease remains a critical priority for global prevention and control strategies [13]. Multiple vaccine platforms are under development, and three live-attenuated formulations have been licensed: Dengvaxia^®^ (Sanofi Pasteur), Qdenga^®^ (Takeda), and Butantan-DV (Instituto Butantan). Although each demonstrates promise, efficacy differs across serotypes and host serostatus, highlighting both the progress achieved and the persistent challenges in attaining broad, durable protection [14]. A recent long-term evaluation of the Butantan dengue vaccine (single-dose regimen) demonstrated a favorable safety profile. Headache, predominantly mild, was the most frequently reported solicited systemic adverse event. Over five years of follow-up, no safety concerns were attributed to vaccination. The overall vaccine efficacy was 65.0% [15]. Similarly, an evaluation of the TAK-003 dengue vaccine in adolescents during a large outbreak in São Paulo, Brazil, predominantly driven by DENV serotypes 1 and 2, showed moderate protection. Adjusted vaccine effectiveness was 50.2% after the first dose and 61.7% after the second dose against symptomatic dengue. Moreover, the first dose conferred 67.5% effectiveness against hospitalization due to dengue [16].

Dengue resurged in the Americas in the late 20th century after decades of decline, with Argentina experiencing increasingly severe outbreaks since the 1990s. In the northern province of Salta, multiple dengue epidemics have been reported involving all four viral serotypes. Early outbreaks included DENV-2 in 1998, DENV-1 in 2002, and DENV-3 in 2003 [17]. Between 2008 and 2014, recurrent epidemics were driven by DENV-1 and DENV-2, with the introduction of DENV-4 documented in 2013–2014 [18,19]. Subsequent outbreaks of greater magnitude occurred in 2016-2018 with DENV-1, in 2019-2020 with co-circulation of DENV-1 and DENV-4, and in 2020-2021 with DENV-1 and DENV-2 [19]. More recently, DENV-2 predominated in 2021-2022, followed by renewed co-circulation of DENV-1 and DENV-2 from 2022 to 2025 [20]. Collectively, these epidemiological records highlight Salta’s vulnerability to sustained transmission and the increased risk of secondary infections.

Considering this epidemiological context, understanding the immunological determinants of disease severity is essential. The objective of this study was to evaluate the influence of acute-phase dengue antibodies on viremia levels and clinical manifestations in a pediatric population (children and adolescents) from the northern province of Salta, Argentina, during the 2016–2018 DENV-1 outbreaks. Specifically, we aimed to assess whether acute-phase antibodies were exerting a potentially pathogenic or protective effect by characterizing antibody profiles, including DENV-reactive IgG, DENV-1-specific IgG, DENV non-structural protein 1 (NS1)-specific IgG, and neutralizing antibodies.

## 2. Materials and Methods

### 2.1. Study Population

This retrospective study included pediatric patients younger than 18 years with laboratory-confirmed DENV infection, residing in the City of San Ramón de la Nueva Orán (also referred to as Orán) and neighboring localities in northwestern Argentina, who sought care at the regional tertiary-level San Vicente de Paul Hospital between 2016 and 2018.

### 2.2. Sample and Data Collection and Dengue Diagnostic Tests

Blood specimens were obtained during the acute phase of illness (≤7 days after symptom onset) for biochemical analyses and for virological and serological assessment of DENV infection. Confirmation of DENV infection was based on the detection of NS1 antigen (Platelia Dengue NS1 Ag, Bio-Rad, Marnes-la-Coquette, France) and IgM antibodies (PanBio Dengue IgM Capture ELISA, Abbott Laboratories, Abbott Park, IL, USA; Dengue IgM Capture ELISA, Instituto Nacional de Enfermedades Virales Humanas [INEVH], Ministry of Health, Argentina) by ELISA. The methodology for IgM detection using capture ELISA (MAC-ELISA, INEVH) has been previously described [21]. DENV serotyping was conducted by reverse transcription followed by polymerase chain reaction (RT-PCR) at the provincial reference laboratory [22].

Data were obtained using standardized clinical-epidemiological surveillance forms for non-specific febrile syndromes and through laboratory analyses conducted during patient care at the San Vicente de Paul Hospital, as previously reported [18,21]. Leukopenia was defined as a leukocyte count < 4000/mm^3^ and thrombocytopenia as a platelet count < 150,000/mm^3^.

Dengue cases were classified as dengue without warning signs (DWoWS), dengue with warning signs, and severe dengue (SD), in accordance with the diagnostic criteria established by the World Health Organization (WHO) [8]. For age-group analysis, stratification was performed according to WHO definitions, distinguishing children (<10 years) from adolescents (≥10 years) [23].

The de-identified patient-level dataset containing demographic, clinical and laboratory assay data used in the analyses is provided in Supplementary Materials.

### 2.3. Determination of DENV Immune Status by Commercial Immunoassay

DENV serostatus was assessed in acute-phase serum samples (≤7 days after symptom onset) using the PanBio Dengue IgG Indirect ELISA (Abbott Diagnostics Korea Inc., Yongin-si, Republic of Korea), according to the manufacturer’s protocol, to detect acute-phase anti-DENV IgG antibodies. Based on these results, DENV serostatus was classified as probable primary or probable secondary infections.

### 2.4. Determination of DENV-Reactive and DENV-1-Reactive IgG Antibodies

IgG antibodies against the four DENV serotypes were quantified in acute-phase serum samples using a laboratory-developed DENV-specific immunoassay [21]. In brief, 96-well plates were coated with a suspension containing equal amounts of the four DENV serotypes (DENV-1 to DENV-4; 1 × 10^5^ plaque-forming units [PFU] of each serotype/mL) in 0.1 M sodium carbonate buffer (pH 9.5) and incubated overnight at 4 °C. DENV-1 Hawaii strain, DENV-2 16,681 strain, DENV-3 H87 strain, DENV-4 8124 strain were used. Wells were then washed three times with PBS containing 0.1% Tween-20 and blocked with 5% skim milk in PBS for 1 h at 37 °C. Two-fold serial dilutions of patient sera (1:100 to 1:12,800) were subsequently added and incubated overnight at 4 °C. Following additional washes, goat anti-human HRP-conjugated IgG (Sigma Aldrich-Merck KGaA, Darmstadt, Germany) was applied for 1 h at 37 °C. After washing, TMB substrate (Invitrogen-Thermo Fisher Scientific, Carlsbad, CA, USA) was added, and the reaction was stopped with 1 M H_2_SO_4_. Optical density (OD) was measured at 450 nm. Titration curves were fitted by non-linear regression to a sigmoidal function using GraphPad Prism v8.4.3, and endpoint titers were defined as the reciprocal of the serum dilution yielding a signal twice the background after subtraction of background signal in all samples. Non-reactive sera (defined as those with endpoint titers < 100) were assigned a titer of 50, corresponding to half of the assay’s limit of detection.

Quantification of IgG antibodies reactive against DENV-1 was performed using the same protocol, except that plates were coated with a viral suspension containing only DENV-1 particles (2 × 10^5^ PFU/mL) in 0.1 M sodium carbonate buffer.

### 2.5. Recombinant NS1 Expression and Purification

Recombinant NS1 was produced using the Bac-to-Bac Baculovirus Expression System (Thermo Fisher Scientific, Carlsbad, CA, USA) according to standard protocols for recombinant baculovirus production. The gene encoding non-structural protein 1 (NS1) of dengue virus serotype 2 (DENV-2, GenBank: U87411.1) was amplified with oligonucleotides 112 (5′-TCCCCCCGGGAATGGATAGTGGTTGCGTTGTGAGC-3′) and 113 (5′-AACTGCAGTTAGTGGTGATGGTGATGATGAGCTGTGACCAAGGAGTTG-3′), and cloned into the transfer vector pFBSD (derived from pFastBac1 [Invitrogen], a kind gift from Oscar Taboga, Instituto de Virología, INTA). The resulting plasmid was transformed into *Escherichia coli* DH10BAC cells to generate a recombinant bacmid DNA carrying NS1-6xHIS construction. Purified bacmid DNA was transfected into Sf9 cells using Polyethylenimine (PEI) reagent to generate recombinant baculovirus.

For protein expression, Sf9 cells were adapted to suspension culture with orbital agitation at 130 rpm and infected at a cell density of 2 × 10^6^ cells/mL at a multiplicity of infection of 3 PFU per cell. The culture supernatant containing NS1-6xHIS was harvested at 6 days post-infection and applied to a Ni-Sepharose high-performance column (GE Healthcare) for protein purification according to the manufacturer’s instructions.

### 2.6. Determination of DENV-NS1-Specific IgG Antibodies

IgG antibodies directed against the DENV NS1 protein were quantified in acute-phase serum samples using a laboratory-developed immunoassay. Ninety-six-well plates were coated with purified recombinant DENV NS1 (1 µg/mL; 100 µL/well) in 0.1 M sodium carbonate buffer (pH 9.5) and incubated overnight at 4 °C. Subsequent steps followed the same protocol used for the detection of anti-DENV antibodies, except that two-fold serial dilutions of patient sera ranging from 1:50 to 1:51,200 were used. As a positive control, pooled immune sera from DENV NS1-immunized mice were used, with detection performed using rabbit anti-mouse HRP-conjugated IgG (Sigma Aldrich-Merck KGaA, Darmstadt, Germany). Non-reactive sera against NS1 (defined as those with endpoint titers < 50) were assigned a titer of 25, corresponding to half of the assay’s limit of detection.

The mouse immunization protocol used to generate DENV NS1-immune control sera is detailed in the Supplementary Methods.

### 2.7. Determination of Neutralizing Antibodies Against DENV-1

Vero cells (ATCC CCL-81) were maintained in MEM (GIBCO, Life Technologies Corporation, Grand Island, NY, USA) supplemented with 10% fetal bovine serum (FBS). The C6/36 mosquito cell line derived from *Aedes albopictus*, adapted to growth at 33 °C, was cultured in Leibovitz’s L-15 medium (GIBCO) supplemented with 0.3% tryptose phosphate broth, 0.02% glutamine, 1% MEM non-essential amino acids, and 10% FBS. The DENV-1 Hawaii strain was propagated in C6/36 cells for 4–6 days, and viral stocks were titrated by plaque assay in Vero cells.

Neutralizing antibodies against DENV-1 were quantified in human sera using a plaque reduction neutralization test (PRNT). Briefly, sera were heat-inactivated at 56 °C for 30 min and subjected to four-fold serial dilutions (1:20 to 1:40,960 in MEM). Each dilution was mixed with 75 PFU of DENV-1 in MEM and incubated at 37 °C for 1 h before inoculation onto confluent Vero cells seeded in 48-well plates. Following a 1 h incubation period at 37 °C, cells were overlaid with MEM containing 2% FBS and 1% methylcellulose and incubated for 7 days at 37 °C. Monolayers were then fixed with 10% formaldehyde, stained with 10% crystal violet, and plaques were counted. Neutralization curves were fitted by non-linear regression to a sigmoidal function, and PRNT_50_ titers were defined as the reciprocal of the serum dilution producing a 50% reduction in plaque numbers relative to controls. Non-neutralizing sera (defined as those with PRNT_50_ values < 20) were given a PRNT_50_ value of 10, corresponding to half of the assay’s limit of detection.

### 2.8. Viremia Quantification

RNA was isolated from patient serum samples using the High Pure Viral RNA Kit (Roche Diagnostics, Mannheim, Germany) following the manufacturer’s protocol. Quantification of viral RNA was performed with the StepOne Real-Time PCR System (Applied Biosystems, Thermo Fisher Scientific Inc., Waltham, MA, USA) employing TaqMan chemistry. Primers and probe were designed to amplify nucleotides 10,589–10,699 within the viral 3′ untranslated region (UTR) [24]. Standard curves were established from 10-fold serial dilutions of viral RNA derived from a purified DENV-1 suspension.

### 2.9. Statistical Analysis

Categorical variables were analyzed using the chi-square test and continuous variables were assessed with the Mann–Whitney test to compare two independent groups. Correlations among study variables were evaluated using Spearman’s correlation coefficient (r_S_). *p*-values from all figures were adjusted for multiple comparisons using the Benjamini–Hochberg false discovery rate (FDR) procedure. *p*-values from tables were adjusted using either the FDR procedure or the Bonferroni correction, as appropriate. Adjusted *p*-values < 0.05 were considered statistically significant. Data were analyzed using Stata/SE version 17.0 for Windows (StataCorp LP).

## 3. Results

### 3.1. Characterization of the Study Population by DENV Immune Status and Age Group

Among 151 pediatric patients with DENV-1 infection who attended San Vicente de Paul Hospital in Orán, Salta Province, Argentina, between 2016 and 2018, the median age was 12 years (interquartile range [IQR] 10–14). Of these, 72 (47.7%) were female and 45 (29.8%) required hospitalization. DWWS was diagnosed in 94 cases (62.3%), while SD occurred in one patient (0.7%) (Table 1). The most common clinical manifestations included fever (100%), myalgia (86.8%), headache (77.5%), arthralgia (72.9%), retroorbital pain (68.9%), leukopenia (62.3%), and nausea (60.9%). The predominant warning signs were abdominal pain (47.7%) and thrombocytopenia (29%). Clinical symptoms stratified by disease severity are presented in Table S1. The demographic and clinical characteristics of the patient with SD are also detailed in Table S1. No evidence of immunocompromise was reported in this patient.

DENV immune status was assessed in patient serum samples collected within seven days of symptom onset to detect antibodies from prior infection. Initially, a commercial indirect immunoassay for DENV IgG was performed to assess the presence of antibodies in acute-phase serum samples. Ninety-nine patients (65.6%) exhibited IgG reactivity above the assay cut-off, suggesting evidence of prior DENV infection (Table 1). Patients with probable secondary infections showed clinical manifestations and laboratory findings comparable to those observed in patients with probable primary infections. Furthermore, no significant association was detected between DENV immune status and disease severity categories.

When stratified by age, adolescents showed elevated hematocrit levels compared with children younger than 10 years (*p* = 0.0018) (Table 2). No statistically significant differences in disease severity or clinical manifestations were observed across age groups.

### 3.2. Correlations of Anti-DENV-, Anti-DENV-1- and Anti-DENV-NS1-Specific IgG Antibodies with Neutralizing Antibodies

To characterize acute-phase DENV antibody profiles in pediatric patients, IgG antibody titers reactive against the four DENV serotypes, DENV-1, and the DENV NS1 protein were quantified using a laboratory-developed immunoassay, and neutralizing antibody levels against DENV-1 were measured by plaque assay. Relative IgG reactivity indexes obtained from the commercial immunoassay, used to assess prior DENV exposure, showed positive correlations with DENV- and DENV-1-reactive IgG antibodies as well as neutralizing antibodies (*p* = 0.0001), but no correlation with DENV NS1-specific antibodies (*p* = 0.3620) (Figure S1A–D). Consistently, probable secondary infections were characterized by significantly higher levels of anti-DENV IgG (*p* = 0.0001), anti-DENV-1 IgG (*p* = 0.0001), and neutralizing antibodies (*p* = 0.0001), whereas anti-NS1 IgG titers did not differ significantly between probable primary and secondary infections (*p* = 0.1404) (Figure S1E–H).

**Figure 1:**
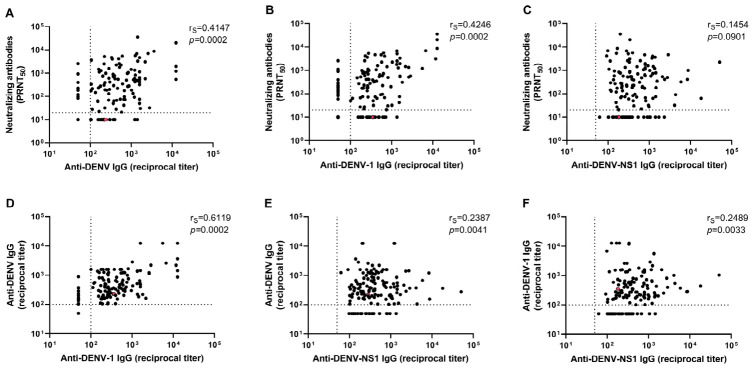
Correlation analyses between acute-phase dengue antibodies, including neutralizing antibodies, anti-DENV, anti-DENV-1, and anti-DENV-NS1 IgG. Shown are correlations between neutralizing DENV antibodies and (**A**) anti-DENV IgG, (**B**) anti-DENV-1 IgG, and (**C**) anti-DENV-NS1 IgG; between anti-DENV IgG titers and (**D**) anti-DENV-1 IgG and (**E**) anti-DENV-NS1 IgG; and between (**F**) anti-DENV-1 IgG and anti-DENV-NS1 IgG. Vertical and horizontal dotted lines indicate assay detection limits. Non-reactive sera were assigned titers corresponding to half the limit of detection. *p*-values were initially calculated with a Spearman correlation test. r_S_, Spearman correlation coefficient. *p*-values were adjusted for multiple comparisons using the Benjamini–Hochberg false discovery rate procedure. Adjusted *p*-values are shown and values < 0.05 were considered significant. Some data points may overlap in the figure, with a single dot representing more than one sample. The patient with SD is highlighted in red.

Neutralizing titers ranged from 21 to 35,604 and correlated positively with both DENV- and DENV-1-reactive IgG levels (r_S_ = 0.4147, *p* = 0.0002, and r_S_ = 0.4246, *p* = 0.0002; Figure 1A,B). In contrast, no association was observed with DENV NS1-specific IgG titers (Figure 1C). DENV-reactive antibodies spanning 103 to 12,449 also correlated positively with DENV-1-reactive antibody levels (endpoint titers ranging from 101 to 12,756) (r_S_ = 0.6119, *p* = 0.0002; Figure 1D). Similarly, DENV NS1-directed antibodies displayed a wide range of titers in this cohort (62 to 50,952) and correlated with both DENV- and DENV-1-reactive antibodies (r_S_ = 0.2387, *p* = 0.0041, Figure 1E, and r_S_ = 0.2489, *p* = 0.0033, Figure 1F, respectively).

### 3.3. Correlation of Acute-Phase Antibodies to DENV and Viremia

**Figure 2:**
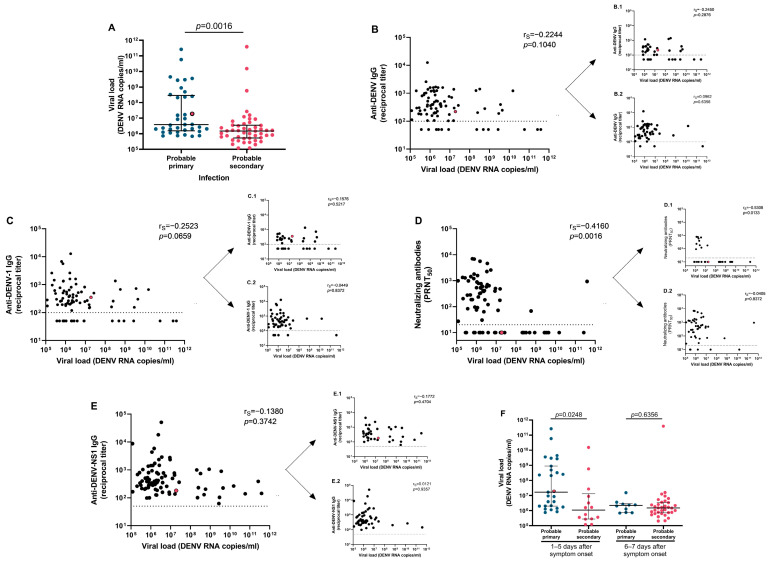
Correlations between acute-phase dengue antibodies and viremia. (**A**) Serum viral load was analyzed according to DENV immune status. Correlations between serum viral load and (**B**) anti-DENV IgG, (**C**) anti-DENV-1 IgG, (**D**) neutralizing DENV antibodies, and (**E**) anti-DENV-NS1 IgG were performed. Stratification by immune status is shown in subpanels 1 and 2: (**B.1**–**E.1**) represent probable primary infections, and (**B.2**–**E.2**) represent probable secondary infections. (**F**) Serum viral load was analyzed according to DENV immune status and stratified by days after symptom onset. (**A**,**F**) Median and interquartile ranges are shown. *p*-values were initially calculated with a Mann–Whitney test. For (**B**–**E**) horizontal dotted lines indicate assay detection limits. Non-reactive sera were assigned titers corresponding to half the limit of detection. *p*-values were initially calculated with a Spearman correlation test. r_S_, Spearman correlation coefficient. (**A**–**F**) All *p*-values were adjusted for multiple comparisons using the Benjamini–Hochberg false discovery rate procedure. Adjusted *p*-values are shown and values <0.05 were considered significant. Some data points may overlap in the figure, with a single dot representing more than one sample. The patient with SD is highlighted in red.

Dengue viremia was assessed across the entire pediatric cohort. A total of 85 patients exhibited detectable DENV RNA titers ranging from 1.2 × 10^5^ to 3.4 × 10^11^ copies/mL. Patients with probable primary infections exhibited 2.6-fold higher serum DENV RNA loads compared to those with probable secondary infections (3.9 × 10^6^ vs. 1.5 × 10^6^ DENV RNA copies/mL; *p* = 0.0016; Figure 2A). No significant correlations were observed between acute-phase anti-DENV, anti-DENV-1, or anti-DENV-NS1 IgG antibodies and viral load (Figure 2B,C,E). In contrast, anti-DENV-1 neutralizing antibodies exhibited a moderate inverse correlation with dengue viremia (Figure 2D). To assess the dependence of neutralizing antibody responses and viral titers on days after symptom onset, correlation analyses were performed. In the study population, antibody responses increased with days of illness, whereas viremia decreased (Figure S2), suggesting that the observed correlations between neutralizing antibodies and viremia may be influenced by the timing of sample collection. When stratifying the cohort by probable primary vs. probable secondary infection, no correlations were observed between antibody responses and viremia (Figure 2B–E, subpanels 1 and 2), except for neutralizing antibodies during probable primary infection, where an inverse correlation with serum viral load was detected (Figure 2D.1). This likely reflects a slight increase in neutralizing capacity, given the low antibody titers observed, beginning toward the end of the ≤7-day window during probable primary infections (Figure S2C.1).

Stratification further revealed that both anti-DENV IgG antibodies and neutralizing antibodies increased with days of illness in probable secondary infections (r_S_ = 0.5038, *p* = 0.0011; Figure S2A.2 and r_S_ = 0.4008, *p* = 0.0097; Figure S2C.2), whereas anti-DENV-1 and anti-DENV-NS1 IgG antibodies did not show a significant rise in probable secondary infections within the ≤7-day window (Figure S2B.2,D.2).

When the analysis was limited to patients with samples collected within ≤5 days of symptom onset, viral load remained higher in probable primary infections compared to probable secondary infections (1.7 × 10^7^ vs. 1.0 × 10^6^ DENV RNA copies/mL; *p* = 0.0248; Figure 2F), and no association between antibody titers and viremia was observed with respect to days of symptoms in this restricted cohort (Figures S3 and S4). Furthermore, no statistically significant differences in viremia levels were detected between probable primary and probable secondary infections at 6–7 days after symptom onset (Figure 2F).

It is important to note that the analysis was restricted to patients with detectable viral titers. Patients with undetectable viremia exhibited antibody titers that were not significantly different from those with detectable viremia (*p* = 0.6249 for anti-DENV IgG, *p* = 0.6249 for anti-DENV-1 IgG, *p* = 0.2436 for neutralizing antibodies, and *p* = 0.6249 for anti-DENV-NS1 IgG; Figure S5). When values corresponding to half the lower limit of detection were assigned to non-detectable viremia samples to include these patients in the analysis, antibody responses did not correlate with viremia levels, with the exception of neutralizing antibodies, which demonstrated an inverse correlation. Stratification of the entire cohort by probable primary vs. probable secondary infection yielded results consistent with those observed in the subset of patients with detectable viremia (Figures S6 and S7).

The demographic characteristics, clinical manifestations and laboratory parameters of patients with detectable viremia levels or of those with samples collected within 5 days after symptom onset are shown in Tables S2, S3 and Tables S4, S5, respectively.

### 3.4. Association of Viremia with Demographic and Clinical Parameters

**Figure 3:**
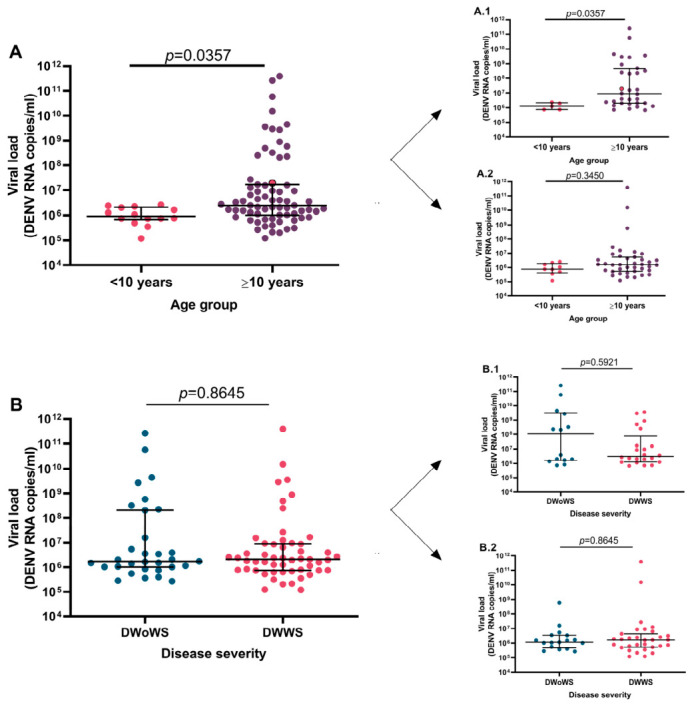
Dengue viremia and its association with demographic and clinical parameters. Serum viral load was analyzed across (**A**) age groups, and (**B**) disease severity categories. Stratification by immune status is shown in subpanels 1 and 2: (**A.1**,**B.1**) represent probable primary infections, and (**A.2**,**B.2**) represent probable secondary infections. Median and interquartile ranges are shown. *p*-values were initially calculated with a Mann–Whitney test. All *p*-values were adjusted for multiple comparisons using the Benjamini–Hochberg false discovery rate procedure. Adjusted *p*-values are shown and values < 0.05 were considered significant. In (**A**,**A.1**,**A.2**) the patient with SD is highlighted in red. DWoWS, dengue without warning signs; DWWS, dengue with warning signs.

To assess the potential association between serum viral load and demographic and clinical features of the disease in pediatric patients, correlation analyses were conducted. Age-stratified analysis (<10 and ≥10 years) indicated that adolescents had higher viral titers than younger children (Figure 3A). When further stratified by infection status, viremia was comparable between the two age groups in probable secondary infections but differed in probable primary infections, with adolescents exhibiting median DENV RNA copy numbers 7.1-fold higher than those of younger children (Figure 3A.1,A.2). The interval from symptom onset to sample collection did not significantly differ between children < 10 and ≥10 years with detectable viremia samples (*p* = 0.5063, Figure S8A). Stratified analyses further showed no association between days from symptom onset and age group in either probable primary or secondary infections (*p* = 0.5063 and *p* = 0.5677, respectively; Figure S8A.1,A.2).

Among the 15 adolescent patients with viremia titers higher than 10^8^ DENV RNA copies/mL, 12 had primary infection and 3 had probable secondary infection (Figure 3A.1,A.2). Within the group of adolescents with primary infection, patients with high viremia exhibited anti-DENV IgG, anti-DENV-1 IgG, and anti-NS1 IgG titers comparable to those with lower viremia (*p* = 0.4443, *p* = 0.6286, and *p* = 0.2674, respectively). In contrast, neutralizing antibody titers were five-fold lower in patients with high viremia compared to those with lower viremia (median 10 [IQR 10–10] vs. 50.8 [IQR 10–390.3], *p* = 0.0116). Among adolescents with probable secondary infection, patients with viremia levels > 10^8^ DENV RNA copies/mL showed anti-DENV IgG, anti-DENV-1 IgG, and neutralizing antibody titers similar to those with lower viremia (*p* = 0.3477, *p* = 0.7467, and *p* = 0.3161, respectively), whereas anti-NS1 IgG titers were approximately two-fold lower in patients with high viremia (median 203.1 [IQR 143.1–266] vs. 411.5 [IQR 281.4–865.6], *p* = 0.0381).

By contrast, viremia did not vary significantly by disease severity, with median values of 1.7 × 10^6^ and 2.1 × 10^6^ RNA copies/mL observed in patients with DWoWS and DWWS, respectively (Figure 3B). The interval from symptom onset to sample collection did not significantly differ between disease severity categories in children with detectable viremia samples (*p* = 0.1689, Figure S8B). When viremia analysis by disease severity (DWoWS vs. DWWS) was stratified by infection status, we found no significant differences in viremia levels according to disease severity within probable primary and probable secondary infections (Figure 3B.1,B.2). Among 8 patients with DWoWS and viremia titers > 10^8^ DENV RNA copies/mL, 7 had probable primary infection and 1 had probable secondary infection. Within both probable primary and probable secondary infection groups, no differences in antibody titers were observed between DWoWS patients with high vs. lower viremia. Similarly, among 7 patients with DWWS and viremia titers > 10^8^ DENV RNA copies/mL, 5 had probable primary infection and 2 had probable secondary infection. Again, no differences in antibody titers were detected between DWWS patients with high vs. lower viremia within each infection group. These results suggest that antibodies may not be controlling viremia levels in these patient groups. The patient diagnosed with SD exhibited a viral load of 1.9 × 10^7^ RNA copies/mL. Additionally, in the pediatric cohort, DENV viremia showed no association with clinical or laboratory features of the disease.

### 3.5. Correlation of Acute-Phase Antibodies to DENV and Age

Analyses were performed to assess the association between acute-phase anti-DENV antibodies and patient age. Stratification by age group revealed no significant differences in acute-phase antibody titers, between children <10 years and adolescents (Figure S9). It is important to note that the interval between symptom onset and sample collection did not differ significantly across age groups (*p* = 0.2531, Figure S10A). Further stratification by probable primary and secondary infection revealed no differences in antibody titers between age groups within either infection category (Figure S9, panels 1 and 2). Within the adolescent group, individuals lacking detectable anti-DENV IgG, anti-DENV-1 IgG, or neutralizing antibodies predominantly had probable primary infections (Figure S9A.1–C.1). Adolescents without detectable anti-DENV or anti-DENV-1 IgG antibodies exhibited viremia levels comparable to those with detectable antibodies across both infection categories, suggesting that these antibodies are not associated with viremia reduction in this population. In contrast, adolescents lacking detectable neutralizing antibodies demonstrated significantly higher viremia levels compared to those with detectable neutralizing antibodies within the probable primary infection group (median 1.1 × 10^8^ DENV RNA copies [IQR 2.2 × 10^6^–1.8 × 10^9^] vs. 2.3 × 10^6^ DENV RNA copies [IQR 1.5 × 10^6^–3.7 × 10^6^]; *p* = 0.0258).

All individuals with antibody levels higher than 10^4^ were undergoing probable secondary infections, likely reflecting boosted antibody responses. Viremia levels in these patients were either undetectable or within the range 7.4 × 10^5^–3.4 × 10^6^ DENV RNA copies/mL.

### 3.6. Association of Acute-Phase Antibodies to DENV and Disease Severity

**Figure 4:**
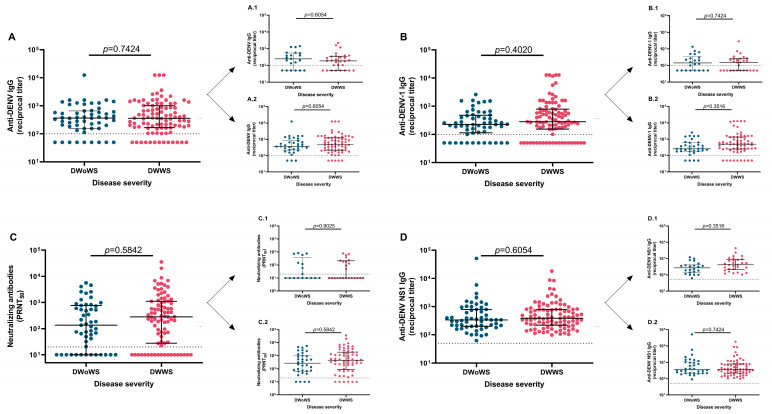
Acute-phase dengue antibodies and disease severity. (**A**) Anti-DENV IgG, (**B**) anti-DENV-1 IgG, (**C**) neutralizing DENV antibodies, and (**D**) anti-DENV-NS1 IgG were analyzed across severity groups. Stratification by immune status is shown in subpanels 1 and 2: (**A.1**–**D.1**) represent probable primary infections, and (**A.2**–**D.2**) represent probable secondary infections. Median and interquartile ranges are shown. *p*-values were initially calculated with a Mann–Whitney test. All *p*-values were adjusted for multiple comparisons using the Benjamini–Hochberg false discovery rate procedure. Adjusted *p*-values are shown and values <0.05 were considered significant. Horizontal dotted lines indicate assay detection limits. Non-reactive sera were assigned titers corresponding to half the limit of detection.

The relationship between acute-phase DENV antibodies and disease severity was assessed in the pediatric cohort (Figure 4). No significant differences were detected for DENV-, DENV-1-, or DENV NS1-specific IgG, nor for neutralizing antibodies across severity categories (DWoWS vs. DWWS) under either infection condition. The interval between symptom onset and sample collection did not differ significantly across severity categories (Figure S10B). It is noteworthy that antibody titers higher than 10^4^ were observed in patients undergoing DWWS (Figure 4A–D). Furthermore, when we stratified the data according to probable primary and probable secondary infection status, we found that individuals without detectable antibody responses exhibited viremia levels that were not significantly different from those with measurable antibodies under either infection condition and severity category. These results suggest that antibody presence was not associated with viremia control in this population. The patient who developed SD was undergoing a probable primary infection, as indicated by a non-reactive PanBio index and a non-reactive neutralizing antibody titer (PRNT_50_ = 10). Consistently, anti-DENV, anti-DENV-1 and anti-DENV-NS1 titers were low in this patient, with values of 219, 352 and 182, respectively.

**Figure 5:**
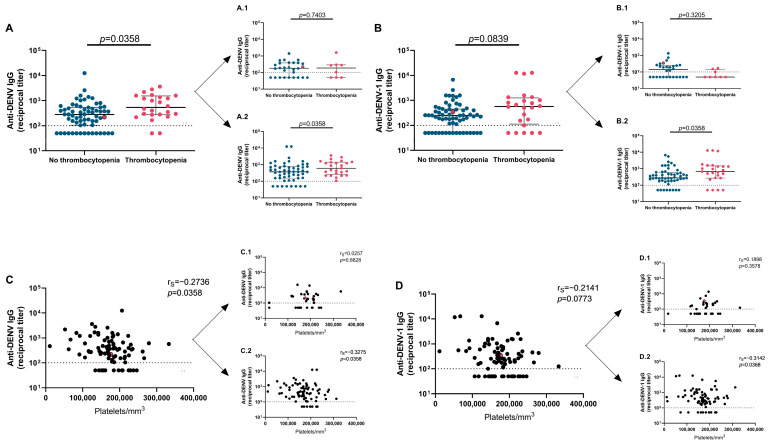
Association between acute-phase dengue antibodies and thrombocytopenia in adolescents. (**A**) Anti-DENV IgG and (**B**) anti-DENV-1 IgG were compared in patients with or without thrombocytopenia. Correlations between platelet counts and (**C**) anti-DENV IgG and (**D**) anti-DENV-1 IgG were performed. Stratification by immune status is shown in subpanels 1 and 2: (**A.1**–**D.1**) represent probable primary infections, and (**A.2**–**D.2**) represent probable secondary infections. (**A**,**B**) Median and interquartile ranges are shown. *p*-values were initially calculated with a Mann–Whitney test. For (**C**,**D**) *p*-values were initially calculated with a Spearman correlation test. r_S_, Spearman correlation coefficient. (**A**–**D**) All *p*-values were adjusted for multiple comparisons using the Benjamin-Hochberg false discovery rate procedure. Adjusted *p*-values are shown and values <0.05 were considered significant. Horizontal dotted lines indicate assay detection limits. Non-reactive sera were assigned titers corresponding to half the limit of detection. Some data points may overlap in the figure, with a single dot representing more than one sample. The patient with SD is highlighted with a distinct color.

We next assessed whether acute-phase DENV antibody levels were linked to the occurrence of clinical symptoms or warning signs. No significant associations were observed for DENV-, DENV-1-, or DENV NS1-specific IgG, nor for neutralizing antibodies in the overall pediatric population. However, stratified analysis by age group (<10 and ≥10 years) revealed that adolescents with thrombocytopenia exhibited higher DENV-reactive IgG titers compared with those without thrombocytopenia (Figure 5A). Stratification by immune status revealed that adolescents with thrombocytopenia undergoing probable secondary infections exhibited DENV- and DENV-1-reactive IgG titers that were 2.4- and 2.3-fold higher, respectively, than those without thrombocytopenia (Figure 5A.2,B.2), whereas no such associations were observed in probable primary infections (Figure 5A.1,B.1). Furthermore, in adolescents with probable secondary infections, DENV- and DENV-1-reactive IgG titers were inversely correlated with platelet counts (r_S_ = −0.3275, *p* = 0.0358; Figure 5C.2, and r_S_ = −0.3142, *p* = 0.0368; Figure 5D.2, respectively). Notably, anti-DENV-NS1 IgG and neutralizing antibody levels did not differ between patients with or without thrombocytopenia, nor did they correlate with platelet counts across the pediatric cohort (Figure S11). Similarly, the interval between symptom onset and sample collection did not differ significantly between adolescents with or without thrombocytopenia and showed no correlation with platelet counts in this patient group (Figure S12).

## 4. Discussion

In this study, we analyzed 151 pediatric patients with DENV-1 infection from the northern province of Salta, Argentina, to investigate the role of acute-phase dengue antibodies in disease outcomes. Patients with probable secondary infections exhibited lower viremia levels compared to those with probable primary infections. Furthermore, in probable secondary infections, antibodies reactive to DENV, DENV-1, and DENV-NS1, as well as neutralizing antibodies, showed no association with viremia. Across clinical severity categories, no significant differences were observed in DENV-, DENV-1-, or DENV NS1-specific IgG, nor in neutralizing antibody levels in both probable primary and probable secondary infections. Age-stratified analysis revealed that adolescents with thrombocytopenia undergoing probable secondary infection displayed increased DENV- and DENV-1-reactive IgG titers compared with those without thrombocytopenia, and these titers were inversely correlated with platelet counts.

In our pediatric cohort, the most frequent clinical manifestations were fever, myalgia, headache, arthralgia, retroorbital pain, leukopenia, and nausea, while the predominant warning signs were abdominal pain and thrombocytopenia. Age-stratified analysis in our cohort revealed that adolescents exhibited lower hematocrit levels compared with children younger than 10 years. In line with our observations, a recent outbreak investigation in Argentina reported that the most common clinical manifestations across the pediatric population included fever, headache, myalgia, and abdominal pain, while laboratory abnormalities comprised leukopenia, thrombocytopenia, and elevated transaminases; notably, adolescents exhibited a greater burden of hematological abnormalities [25]. Similarly, a study conducted in Colombia reported that older children exhibited elevated hematocrit values and additional hematological alterations compared with younger children [26].

In our pediatric cohort, children with probable secondary DENV infections showed elevated levels of anti-DENV IgG, anti-DENV-1 IgG, and neutralizing antibodies, while exhibiting comparable anti-DENV-NS1 IgG titers relative to those with probable primary infections. Furthermore, patients with probable primary infections exhibited DENV viremia levels higher than those with probable secondary infections. Similar results have been reported previously [27,28,29,30]. Potential explanations for this observation include several possibilities. First, DENV RNA was measured in serum, while viral particles may be sequestered in other tissues, thereby escaping detection in circulation. Another possibility is that, in probable secondary infections, transiently elevated viremia early in disease progression, potentially influenced by ADE, could trigger stronger immune responses, shifting the viremia curve to the left and resulting in an earlier viral peak [27,28]. In our study population, the relatively small number of patients sampled within 1–2 days of symptom onset may have limited our ability to capture peak viremia in probable secondary infections, as serum viral loads may already have begun to decline by the time of measurement. Conversely, in probable primary infections, peak viremia may occur later in the course of illness, with the viremia curve shifted to the right [27,28].

In our study, stratified analysis by infection status revealed no significant correlations between antibody responses and viremia in probable secondary infections. Furthermore, analysis at both the individual and group levels revealed that acute-phase antibody titers were not associated with viremia control in this pediatric cohort. These findings suggest that additional factors, likely immune cell activity, may contribute to the reduced viremia observed in probable secondary relative to probable primary infections. T cells have been shown to play a role in viral clearance during dengue infections. In acute dengue, early DENV-specific T cell responses have been associated with lower viremia and milder disease, supporting their protective role in viral control [31,32]. Natural killer (NK) cells are also implicated in the early antiviral response to dengue, as activated NK cell populations expand during the acute phase and correlate with favorable clinical outcomes [33,34]. Together, T cells and NK cells are likely among the earliest cellular responders contributing to dengue viral clearance, which generally occurs within the first week after symptom onset [32]. In contrast, robust humoral responses-particularly neutralizing antibody responses- typically develop later and are most commonly evaluated around day 28 after infection [35].

Our study found that anti-DENV-NS1 antibody titers were comparable between probable primary and secondary infections within the cohort. In contrast, previous reports have indicated that secondary infections are associated with higher anti-NS1 responses and an inverse relationship between anti-DENV-NS1 antibody titers and circulating NS1 antigen levels, supporting the formation of NS1 antigen–antibody immune complexes and implicating these antibodies in NS1 clearance [36,37].

In our study, viremia was not associated with disease severity in pediatric patients. Similar findings have been reported in other pediatric cohorts, where DENV load and NS1 antigenemia generally do not predict clinical severity, though they may influence recovery dynamics and depend on immune status [38,39]. Evidence from a study including both pediatric and adult patients also showed no link between viral load and disease severity but highlighted a relationship with the infecting DENV serotype [40]. In contrast to our findings, several investigations have reported positive associations between higher DENV viremia and disease severity [27,28,41,42,43]. Elevated viral loads have been linked to secondary infection status, more severe thrombocytopenia, and greater hemoconcentration, with viral kinetics varying by immune status and viral serotype [28,42]. Moreover, higher plasma viremia has been associated with severe dengue and plasma leakage, independent of DENV serotype or immune status [27]. Kinetic studies further indicated that elevated viremia was correlated with subsequent decreases in platelet counts and an increased risk of severe dengue and plasma leakage [43]. Early high viremia has also been associated with progression to severe dengue, suggesting its potential utility as a prognostic marker [41]. In our cross-sectional study, viremia was assessed only once after symptom onset, restricting our conclusions to that single measurement and limiting the ability to characterize the temporal dynamics of viremia in relation to disease severity.

In our cohort, DENV viremia levels did not differ significantly between age groups in probable secondary infections, whereas adolescents with probable primary DENV infection exhibited higher viremia compared with younger children. This finding should be interpreted with caution, given the relatively small number of children younger than 10 years included in this subgroup analysis. Previous research has identified age as an important determinant of dengue clinical manifestations, potentially influencing disease expression through differences in host susceptibility and immune history [44]. Although age-dependent differences in immune responses and viral clearance could plausibly contribute to the elevated viral loads observed in adolescents [45,46], our study was not designed to investigate the underlying mechanisms. Therefore, larger studies with a more balanced age distribution will be required to determine whether the observed differences reflect true biological variation or are partly attributable to sample size limitations.

Acute-phase dengue antibody titers in our pediatric cohort were comparable between children younger than 10 years and adolescents, in either probable primary or probable secondary infections. Age has been reported as an important determinant of preexisting dengue antibody profiles in pediatric populations, reflecting both immune maturation and cumulative viral exposure [47,48,49]. Antibody prevalence and titers generally increase with age due to repeated dengue infections, resulting in higher seroprevalence among older children [47]. In contrast, in infants, maternally derived antibodies progressively decline during the first year of life, creating a transient window of susceptibility when antibody concentrations may fall to subneutralizing levels [50,51]. Age may therefore influence dengue immune responses by shaping antibody titers, neutralization breadth, enhancement risk, and immune maturation.

In our study, no significant correlation was detected between acute-phase dengue antibody profiles and disease severity. The relationship between preexisting dengue antibodies and disease severity in children is complex, reflecting both quantitative and qualitative features of the humoral immune response. Low to intermediate antibody levels, particularly subneutralizing titers, have been implicated in increased risk of severe disease through ADE during secondary infections [52,53]. In contrast, high titers of neutralizing antibodies and broadly cross-neutralizing responses are generally associated with protection [52,54] and a lower incidence of severe clinical outcomes [55]. However, these associations are not consistent across studies, and mixed findings have been reported [55,56]. A recent study found no significant difference in severity between primary and secondary infections in pediatric cohorts from India [56]. Such heterogeneity may be influenced by factors including the infecting dengue virus serotype [55,57], the concentration and functional properties of preexisting antibodies [52,54], and the interval since prior infection, which shapes antibody maturation and waning [52]. In our study, data on the time interval between prior and current infections were unavailable for the patients, which may have influenced our results. Furthermore, the outbreak under study involved patients infected with DENV-1, with no evidence of co-circulation of other serotypes in the region. Collectively, these findings emphasize that preexisting dengue immunity does not uniformly predict disease severity in pediatric populations but instead reflects a dynamic interplay between protective and pathogenic immune mechanisms.

In our study population, thrombocytopenia and platelet counts did not differ significantly according to DENV immune status. However, among adolescents experiencing secondary infections, the presence of anti-DENV and anti-DENV-1 antibodies inversely correlated with platelet counts. Secondary infection in the presence of cross-reactive antibodies has been associated with enhanced thrombocytopenia through mechanisms such as ADE, platelet-associated IgG formation, and immune-mediated platelet destruction [58,59,60]. ADE promotes viral replication, while immune complexes contribute to platelet damage and vascular endothelial injury, thereby facilitating hemorrhagic manifestations [59].

Platelet counts typically decline during peak dengue viremia, showing inverse correlations with viral load and dengue-specific antibody responses [58,61,62]. While DENV infection can impair platelet production through megakaryocyte infection and promote platelet activation and apoptosis [63], our pediatric cohort did not demonstrate correlations between platelet counts and viremia.

Anti-NS1 antibodies have been reported to cross-react with platelet antigens, thereby inhibiting platelet aggregation [64]. Through molecular mimicry, anti-NS1 antibodies can also opsonize platelets and promote their clearance by macrophages via Fcγ receptor interactions [24]. In our cohort, no correlation between anti-NS1 antibodies and platelet counts nor an association with thrombocytopenia was observed.

The association between total DENV- and DENV-1-reactive IgG levels and platelet count in adolescents with probable secondary infections, in contrast to the absence of correlation with neutralizing antibodies or anti-DENV-NS1 IgG, likely reflects functional heterogeneity among antibody populations. Total DENV-reactive IgG represents a diverse repertoire of IgG directed against multiple viral antigens (including E and prM), some of which may contribute to immune complex formation or other Fcγ receptor-mediated processes possibly implicated in platelet depletion. By comparison, neutralizing antibodies primarily mediate antiviral activity and may not be necessarily involved in platelet interactions. Although anti-NS1 antibodies have been reported to cross-react with platelet-associated molecules and impair platelet function, the total anti-NS1 IgG measurements in our study do not distinguish potentially pathogenic antibodies from non-pathogenic ones. These differences may potentially account for the distinct associations observed with platelet counts.

Our study has several limitations. Probable secondary infections were classified based on the detection of DENV-reactive IgG in acute-phase serum using a commercial immunoassay. Alternative approaches to assess immune status include determination of the IgM/IgG ratio by immunoassay, plaque reduction neutralization tests, and evaluation of IgG in paired acute-convalescent samples, which can provide further evidence of prior exposure to DENV [65,66]. In our study, PRNT was performed specifically against DENV-1 to assess neutralization of the infecting serotype. Extending neutralization assays to the other three serotypes would have offered a broader perspective on the neutralizing capacity of acute-phase antibodies and provided additional confirmation of secondary infections within the cohort. Importantly, our approach of assessing IgG antibody detection in serum samples collected within 7 days of symptom onset partially reflects antibody profiles influenced by the ongoing infection, rather than exclusively representing preexisting antibodies. Anti-DENV-NS1 antibodies were measured using recombinant NS1 protein from DENV-2; however, NS1 exhibits substantial conservation across serotypes, with approximately 60–80% amino acid sequence homology among DENV-1-4. An additional limitation was the assignment of values below the assay detection limit as half the lower limit of detection for statistical analysis. Although widely applied in immunological and virological studies, this approach may affect the distribution of low-range values and influence statistical associations. For antibody determinations, the proportion of samples below the detection threshold was relatively modest and depended on the method used. However, for dengue viremia, given the number of samples below the detection threshold, this approach was only used for comparison between patients with detectable or non-detectable viremia, due to the possibility that this data handling approach influenced the observed associations.

Reciprocal titers of anti-DENV IgG, anti-DENV-1 IgG, and anti-DENV-NS1 IgG were determined using in-house immunoassays. Anti-DENV IgG and anti-DENV-1 IgG levels correlated well with PanBio indexes obtained from the commercial ELISA (*p* = 0.0001), which was used to differentiate probable primary from probable secondary infections. However, probable background signals were observed with the in-house assays, resulting in antibody titers in probable primary infections above the detection limit. A slight increase in antibody titers was also noted in probable primary infection samples collected 6–7 days after symptom onset. Taken together, the performance of the in-house anti-DENV IgG, anti-DENV-1 IgG, and anti-DENV-NS1 IgG immunoassays, in combination with neutralization assays, provided distinct antibody profiles across patients.

Furthermore, we acknowledge that the sample size available for the analyses, particularly after stratification according to probable primary and probable secondary DENV infection status, resulted in relatively small subgroup sizes. Consequently, the statistical power to detect associations between acute-phase antibodies and disease severity, or between viremia and disease severity, may have been reduced. Moreover, the potential role of anti-DENV and anti-DENV-1 IgG antibodies in platelet depletion was inferred from inverse correlations with platelet counts. Definitive confirmation of this effect would require functional assays such as platelet aggregation studies, apoptosis assays, and detection of platelet-antibody immune complexes. Likewise, in vitro ADE assays were not performed, limiting direct evidence that cross-reactive non-neutralizing antibodies can enhance DENV infection.

## 5. Conclusions

In our pediatric cohort, probable secondary DENV infections were characterized by the presence of acute-phase antibodies that were not associated with viremia control. These antibodies may facilitate immune complex formation and contribute to platelet depletion. In adolescents, profiles of anti-DENV and anti-DENV-1 IgG antibodies were associated with reduced platelet counts. These findings highlight the critical balance between protective and pathogenic roles of acute-phase antibodies in dengue immunopathogenesis in children and underscore important implications for vaccine design. Effective vaccines must elicit robust neutralizing responses while minimizing cross-reactivity and the risk of ADE.

## Figures and Tables

**Table 1:** Demographic characteristics, clinical manifestations and laboratory parameters of the study population by DENV immune status.

Characteristics	Total	Probable Primary Infection	Probable Secondary Infection	*p*
Demographics				
Number of patients, *n* (%)	151 (100)	52 (34.4)	99 (65.6)	-
Age group, *n* (%)				0.6440
<10 years	32 (21.2)	7 (13.5)	25 (25.3)	-
≥10 years	119 (78.8)	45 (86.5)	74 (74.7)	-
Sex, *n* (%)				0.9385
Female	72 (47.7)	24 (46.2)	48 (48.5)	-
Male	79 (52.3)	28 (53.8)	51 (51.5)	-
Disease severity, *n* (%)				0.6440
DWoWS	56 (37.1)	22 (42.3)	34 (34.3)	-
DWWS	94 (62.3)	29 (55.8)	65 (65.7)	-
SD	1 (0.7)	1 (1.9)	0 (0.0)	-
Hospitalized, *n* (%)	45 (29.8)	15 (28.8)	30 (30.3)	0.9385
Clinical manifestations				
Constitutional, *n* (%)				
Fever	151 (100)	52 (100)	99 (100)	-
Headache	117 (77.5)	40 (76.9)	77 (77.8)	0.9385
Retroorbital pain	104 (68.9)	43 (82.7)	61 (61.6)	0.2240
Bilateral eyelid edema	2 (1.3)	0 (0.0)	2 (2.0)	0.7047
Conjunctival injection	68 (45.0)	23 (44.2)	45 (45.5)	0.9385
Musculoskeletal, *n* (%)				
Myalgia	131 (86.8)	48 (92.3)	83 (83.8)	0.6440
Arthralgia	110 (72.9)	41 (78.9)	69 (69.7)	0.6440
Cutaneous, *n* (%)				
Rash	61 (40.4)	20 (38.5)	41 (41.4)	0.9385
Pruritus	39 (25.8)	15 (28.9)	24 (24.2)	0.9385
Gastrointestinal, *n* (%)				
Nausea	92 (60.9)	30 (57.7)	62 (62.6)	0.9385
Vomiting	55 (36.4)	17 (32.7)	38 (38.4)	0.9385
Diarrhea	36 (23.8)	11 (21.2)	25 (25.3)	0.9385
Abdominal pain	72 (47.7)	24 (46.2)	48 (48.5)	0.9385
Hepatomegaly	2 (1.3)	1 (1.9)	1 (1.0)	0.9385
Splenomegaly	2 (1.3)	1 (1.9)	1 (1.0)	0.9385
Jaundice	5 (3.3)	2 (3.9)	3 (3.0)	0.9385
Oligoanuria	1 (0.7)	1 (1.9)	0 (0.0)	0.6440
Respiratory, *n* (%)				
Cough	35 (23.2)	10 (19.2)	25 (25.3)	0.8723
Dyspnea	8 (5.3)	1 (1.9)	7 (7.1)	0.6440
Tachypnea	3 (2.0)	0 (0.0)	3 (3.0)	0.6440
Neurological, *n* (%)				
Confusional syndrome	1 (0.7)	1 (1.9)	0 (0.0)	0.6440
Meningeal syndrome	1 (0.7)	1 (1.9)	0 (0.0)	0.6440
Hemorrhagic syndrome	6 (4.0)	2 (3.9)	4 (4.0)	0.9540
Laboratory findings				
Leukopenia, *n*/N (%)	71/114 (62.3)	24/38 (63.2)	47/76 (61.8)	0.9385
Thrombocytopenia, *n*/N (%)	33/114 (29.0)	8/36 (22.2)	25/78 (32.1)	0.7047
Hematocrit (%), median (IQR)	37 (35–39)	37 (36–39)	37 (35–39)	0.9999
Leukocytes/mm^3^, median (IQR)	3560 (2650–4900)	3565 (2700–4890)	3560 (2590–5125)	0.9999
Platelets ×1000/mm^3^, median (IQR)	173 (142–206)	184 (152–212)	169 (142–202)	0.6768

*p*-values were initially calculated with Pearson’s χ^2^ test for categorical variables and a Mann–Whitney test for continuous variables. To account for multiple comparisons, *p*-values for categorical variables were adjusted using the Benjamini–Hochberg false discovery rate procedure, while those for continuous variables were adjusted using the Bonferroni correction. Adjusted *p*-values are reported, with values < 0.05 considered statistically significant. Abbreviations: IQR, interquartile range; DWoWS, dengue without warning signs; DWWS, dengue with warning signs; SD, severe dengue.

**Table 2:** Demographic characteristics, clinical manifestations and laboratory parameters of the study population by age group.

Characteristics	<10 Years	≥10 Years	*p*
Demographics			
Number of patients, *n* (%)	32 (21.2)	119 (78.8)	-
Sex, *n* (%)			0.1980
Female	21 (65.6)	51 (42.9)	-
Male	11 (34.4)	68 (57.1)	-
Severity categories, *n* (%)			0.0945
DWoWS	4 (12.5)	52 (43.7)	-
DWWS	28 (87.5)	66 (55.5)	-
SD	0 (0.0)	1 (0.8)	-
Hospitalized, *n* (%)	9 (28.1)	36 (30.3)	0.8802
Clinical manifestations			
Constitutional, *n* (%)			
Fever	32 (100)	119 (100)	-
Headache	24 (75.0)	93 (78.2)	0.8802
Retroorbital pain	19 (59.4)	85 (71.4)	0.6426
Bilateral eyelid edema	1 (3.1)	1 (0.8)	0.6563
Conjunctival injection	17 (53.1)	51 (42.9)	0.6563
Musculoskeletal, *n* (%)			
Myalgia	25 (78.1)	106 (89.1)	0.4725
Arthralgia	19 (59.4)	91 (76.5)	0.2916
Cutaneous, *n* (%)			
Rash	13 (40.6)	48 (40.3)	0.9760
Pruritus	13 (40.6)	26 (21.9)	0.2093
Gastrointestinal, *n* (%)			
Nausea	17 (53.1)	75 (63.0)	0.6563
Vomiting	13 (40.6)	42 (35.3)	0.8141
Diarrhea	8 (25.0)	28 (23.5)	0.8952
Abdominal pain	22 (68.3)	50 (42.0)	0.0945
Hepatomegaly	0 (0.0)	2 (1.7)	0.8141
Splenomegaly	0 (0.0)	2 (1.7)	0.8141
Jaundice	0 (0.0)	5 (4.2)	0.6426
Oligoanuria	0 (0.0)	1 (0.8)	0.8141
Respiratory, *n* (%)			
Cough	8 (25.0)	27 (22.7)	0.8802
Dyspnea	2 (6.3)	6 (5.0)	0.8802
Tachypnea	1 (3.1)	2 (1.7)	0.8141
Neurological, *n* (%)			
Confusional syndrome	0 (0.0)	1 (0.8)	0.8141
Meningeal syndrome	0 (0.0)	1 (0.8)	0.8141
Hemorrhagic syndrome	1 (3.1)	5 (4.2)	0.8802
Laboratory findings			
Leukopenia. *n*/N (%)	14/27 (51.9)	57/87 (65.5)	0.6426
Thrombocytopenia. *n*/N (%)	10/26 (38.5)	23/88 (26.1)	0.6426
Hematocrit (%), median (IQR)	36 (33–37)	38 (36–40)	0.0018
Leukocytes/mm^3^, median (IQR)	3860 (3010–5530)	3400 (2580–4900)	0.7578
Platelets ×1000/mm^3^, median (IQR)	165 (126–219)	177 (147–206)	0.9999

*p*-values were initially calculated with Pearson’s χ^2^ test for categorical variables and a Mann–Whitney test for continuous variables. To account for multiple comparisons, *p*-values for categorical variables were adjusted using the Benjamini–Hochberg false discovery rate procedure, while those for continuous variables were adjusted using the Bonferroni correction. Adjusted *p*-values are reported, with values < 0.05 considered statistically significant. Abbreviations: IQR, interquartile range; DWoWS, dengue without warning signs; DWWS, dengue with warning signs; SD, severe dengue.

## Data Availability

The original contributions presented in this study are included in the article/Supplementary Materials. Further inquiries can be directed to the corresponding author.

## Supplementary Materials

The following supporting information can be downloaded at: https://www.mdpi.com/article/10.3390/v18070741/s1; Database Dengue Oran; Supplementary Methods: Experimental Animals and Ethics Statement, Immunization protocol to produce anti-NS1 polyclonal control sera; Table S1: Demographic characteristics, clinical manifestations and laboratory parameters of the study population by dengue disease severity; Table S2: Demographic characteristics, clinical manifestations and laboratory parameters of patients with detectable viremia, stratified by DENV immune status; Table S3: Demographic characteristics, clinical manifestations and laboratory parameters of patients with detectable viremia, stratified by age; Table S4: Demographic characteristics, clinical manifestations and laboratory parameters of patients with samples collected within 5 days after symptom onset, stratified by DENV immune status; Table S5: Demographic characteristics, clinical manifestations and laboratory parameters of patients with samples collected within 5 days after symptom onset, stratified by age; Figure S1: Correlation analyses between acute-phase dengue antibody titers and relative dengue IgG reactivity indexes; Figure S2: Correlations between timing of sample collection and acute-phase dengue antibodies or viremia in patients with detectable viremia levels; Figure S3: Correlations between acute-phase dengue antibodies and viremia in patients with detectable viremia levels and samples collected within 5 days after symptom onset; Figure S4: Correlations between timing of sample collection and acute-phase dengue antibodies or viremia in patients with detectable viremia levels and samples collected within 5 days after symptom onset; Figure S5: Acute-phase dengue antibody titers in patients with detectable or undetectable viremia; Figure S6: Correlations between acute-phase dengue antibodies and viremia in the overall study population; Figure S7: Correlations between timing of sample collection and acute-phase dengue antibodies or viremia in the overall study population; Figure S8: Timing of sample collection by age group and disease severity in patients with detectable viremia levels; Figure S9: Acute-phase dengue antibodies and patient age; Figure S10: Timing of sample collection by age group and disease severity in the overall study population; Figure S11: Association between acute-phase dengue antibodies and thrombocytopenia in adolescents; Figure S12: Timing of sample collection and thrombocytopenia in adolescents.

## Abbreviations

The following abbreviations are used in this manuscript:

ADE	antibody-dependent enhancement
DENV	dengue virus
DENV-1-4	dengue virus serotypes 1-4
DWoWS	dengue without warning signs
DWWS	dengue with warning signs
FBS	fetal bovine serum
HRP	horseradish peroxidase
IQR	interquartile range
MAC-ELISA	IgM antibody capture enzyme-linked immunosorbent assay
MEM	minimum essential medium
NS1	non-structural protein 1
NS1-6xHIS	non-structural protein 1 with hexahistidine tag
PFU	plaque-forming units
PRNT	plaque reduction neutralization test
RT-PCR	reverse transcription polymerase chain reaction
SD	severe dengue
TMB	3,3′,5,5′-tetramethylbenzidine
