# Supplementary material for: Acute-Phase Dengue Antibody Profiles in Pediatric Patients: Influence on Viremia and Disease Manifestations
Source: Viruses. 2026 Jul 3;18(7):741. doi: 10.3390/v18070741 (PMC13431624; doi:10.3390/v18070741)
Supplement: Supplementary file 1 [file viruses-18-00741-s001.zip › Supplementary Methods.pdf]

Supplementary Materials and Methods

1.1. Experimental Animals and Ethics Statement

Animals were maintained under standard laboratory conditions with free access to food and water. Mice were anesthetized with isoflurane before sacrifice by cervical dislocation by trained personnel, and all efforts were made to minimize their suffering.

The experimental protocol was approved by the Committee on the Ethics of Animal Experiments of the Universidad de San Martín (Resolution No. 07/2020) and adhered to the ARRIVE guidelines.

1.2. Immunization protocol to produce anti-NS1 polyclonal control sera

The immunization protocol was designed to induce the production of anti-NS1 antibodies for subsequent serological assays. Five 8-week-old male BALB/c mice were immunized intraperitoneally with recombinant NS1 antigen emulsified with aluminum hydroxide as an adjuvant. Following an initial immunization dose of 20 µg of NS1 (200 µl total volume), two 10 µg boosters were administered at 20-day intervals. At the end of the immunization schedule, blood samples were collected and sera were obtained by centrifugation and stored for subsequent serological assays. The mice were euthanized by cervical dislocation.
