# Supplementary material for: Acute-Phase Dengue Antibody Profiles in Pediatric Patients: Influence on Viremia and Disease Manifestations
Source: Viruses. 2026 Jul 3;18(7):741. doi: 10.3390/v18070741 (PMC13431624; doi:10.3390/v18070741)
Supplement: Supplementary file 1 [file viruses-18-00741-s001.zip › Supplementary Tables S1-S5 and Figures S1-S12.pdf]

Supplementary Tables and Figures

Table S1. Demographic characteristics, clinical manifestations and laboratory parameters of the study population by dengue disease severity.

**Characteristics**	**DWoWS**	**DWWS**	**SD**	***p***
Demographics				
Number of patients, *n* (%)	56 (37.1)	94 (62.3)	1 (0.7)	-
Age in years, median (IQR)	13 (11-15)	12 (9-14)	13	0.4860
Age range in years	6-17	1-17	13	-
Age group, *n* (%)				0.0280
<10 years	4 (7.1)	28 (29.8)	0 (0.0)	-
≥10 years	52 (92.9)	66 (70.2)	1 (100)	-
Sex, *n* (%)				0.4860
Female	22 (39.3)	50 (53.2)	0 (0.0)	-
Male	34 (60.7)	44 (46.8)	1 (100)	-
Hospitalized, *n* (%)	16 (28.6)	28 (29.8)	1 (100.0)	0.6867
Clinical manifestations				
Constitutional, *n* (%)				
Fever	56 (100)	94 (100)	1 (100)	-
Headache	43 (76.8)	73 (77.7)	1 (100.0)	0.9472
Retroorbital pain	43 (76.8)	60 (63.8)	1 (100.0)	0.5303
Bilateral eyelid edema	0 (0.0)	2 (2.1)	0 (0.0)	- (*)
Conjunctival injection	27 (48.2)	41 (43.6)	0 (0.0)	0.7481
Musculoskeletal, *n* (%)				
Myalgia	51 (91.1)	79 (84.0)	1 (100.0)	0.7027
Arthralgia	43 (76.8)	66 (70.2)	1 (100.0)	0.7481
Cutaneous, *n* (%)				
Rash	22 (39.3)	39 (41.5)	0 (0.0)	0.8003
Pruritus	11 (19.6)	28 (29.8)	0 (0.0)	0.6867
Gastrointestinal, *n* (%)				
Nausea	36 (64.3)	55 (58.5)	1 (100.0)	0.7481
Vomiting	20 (35.7)	34 (36.2)	1 (100.0)	0.7027
Diarrhea	8 (14.3)	28 (29.8)	0 (0.0)	0.4410
Abdominal pain	0 (0.0)	72 (76.6)	0 (0.0)	- (*)
Hepatomegaly	0 (0.0)	1 (1.1)	1 (100.0)	- (*)
Splenomegaly	1 (1.8)	0 (0.0)	1 (100.0)	0.0011
Jaundice	4 (7.1)	1 (1.1)	0 (0.0)	0.4860
Oligoanuria	0 (0.0)	0 (0.0)	1 (100.0)	0.0011
Respiratory, *n* (%)				
Cough	11 (19.6)	24 (25.5)	0 (0.0)	0.7535
Dyspnea	3 (5.4)	5 (5.3)	0 (0.0)	0.9790
Tachypnea	1 (1.8)	2 (2.1)	0 (0.0)	0.9790
Neurological, *n* (%)				
Confusional syndrome	0 (0.0)	0 (0.0)	1 (100.0)	- (*)
Meningeal syndrome	0 (0.0)	0 (0.0)	1 (100.0)	- (*)
Hemorrhagic syndrome	0 (0.0)	5 (5.3)	1 (100.0)	- (*)
Laboratory findings				

Leukopenia, *n*/N (%)	21/38 (55.3)	49/75 (65.3)	1/1 (100.0)	0.7027
Thrombocytopenia, *n*/N (%)	0/38 (0.0)	33/75 (44.0)	0/1 (0.0)	- (*)
Hematocrit (%), median (IQR)	37 (35-41)	37 (35-39)	35	0.9999
Leukocytes/mm^3, median (IQR)	3760 (2580-6000)	3500 (2700-4790)	3380	0.9999
Platelets ×1000/mm^3, median (IQR)	195 (171-223)	161 (122-188)	176	0.0006

p-values were initially calculated using Pearson's χ^2 test for categorical variables and the Kruskal–Wallis test for continuous variables. To account for multiple comparisons, *p*-values for categorical variables were adjusted using the Benjamini–Hochberg false discovery rate procedure, while those for continuous variables were adjusted using the Bonferroni correction. Adjusted *p*-values are reported, with values <0.05 considered statistically significant. Abbreviations: DWoWS, dengue without warning signs; DWWS, dengue with warning signs; SD, severe dengue; IQR, interquartile range. (*) considered within DWWS or SD definitions.

Supplementary Tables and Figures

Table S2. Demographic characteristics, clinical manifestations and laboratory parameters of patients with detectable viremia, stratified by DENV immune status.

**Characteristics**	**Total**	**Probable Primary infection**	**Probable Secondary infection**	***p***
Demographics				
Number of patients, *n* (%)	85 (100)	37 (43.5)	48 (56.5)	-
Age group, *n* (%)				0.9083
<10 years	14 (16.5)	5 (13.5)	9 (18.8)	-
≥10 years	71 (83.5)	32 (86.5)	39 (81.3)	-
Sex, *n* (%)				0.9083
Female	38 (44.7)	15 (40.5)	23 (47.9)	-
Male	47 (55.3)	22 (59.5)	25 (52.1)	-
Disease severity, *n* (%)				0.9083
DWoWS	31 (36.5)	14 (37.8)	17 (35.4)	-
DWWS	53 (62.4)	22 (59.5)	31 (64.6)	-
SD	1 (1.2)	1 (2.7)	0 (0)	-
Hospitalized, *n* (%)	26 (30.6)	11 (29.7)	15 (31.3)	0.9300
Clinical manifestations				
Constitutional, *n* (%)				
Fever	85 (100)	37 (100)	48 (100)	-
Headache	65 (76.5)	28 (75.7)	37 (77.1)	0.9300
Retroorbital pain	61 (71.8)	29 (78.4)	32 (66.7)	0.6975
Bilateral eyelid edema	2 (2.4)	0 (0)	2 (4.2)	0.6975
Conjunctival injection	35 (41.2)	16 (43.2)	19 (39.6)	0.9300
Musculoskeletal, *n* (%)				
Myalgia	77 (90.6)	34 (91.9)	43 (89.6)	0.9300
Arthralgia	67 (78.8)	29 (78.4)	38 (79.2)	0.9300
Cutaneous, *n* (%)				
Rash	29 (34.1)	12 (32.4)	17 (35.4)	0.9300
Pruritus	18 (21.2)	10 (27.0)	8 (16.7)	0.6975
Gastrointestinal, *n* (%)				
Nausea	50 (58.8)	22 (59.5)	28 (58.3)	0.9300
Vomiting	23 (27.1)	9 (24.3)	14 (29.2)	0.9300
Diarrhea	22 (25.9)	10 (27.0)	12 (25.0)	0.9300
Abdominal pain	44 (51.8)	21 (56.8)	23 (47.9)	0.9025
Hepatomegaly	1 (1.2)	1 (2.7)	0 (0)	0.6975
Splenomegaly	2 (2.4)	1 (2.7)	1 (2.1)	0.9300
Jaundice	3 (3.5)	2 (5.4)	1 (2.1)	0.9025
Oligoanuria	1 (1.2)	2 (2.7)	0 (0)	0.6975
Respiratory, *n* (%)				
Cough	17 (20.0)	8 (21.6)	9 (18.8)	0.9300
Dyspnea	3 (3.5)	0 (0)	3 (6.3)	0.6975
Tachypnea	2 (2.4)	0 (0)	2 (4.2)	0.6975
Neurological, *n* (%)				
Confusional syndrome	1 (1.2)	1 (2.7)	0 (0)	0.6975
Meningeal syndrome	1 (1.2)	1 (2.7)	0 (0)	0.6975
Hemorrhagic syndrome	5 (5.9)	1 (2.7)	4 (8.3)	0.6975
Laboratory findings				
Leukopenia, *n*/N (%)	43/61 (70.5)	20/27 (74.1)	23/34 (67.7)	0.9300
Thrombocytopenia, *n*/N (%)	13/60 (21.7)	2/26 (7.7)	11/34 (32.4)	0.6160
Hematocrit (%), median (IQR)	37 (36-40)	37 (37-40)	38 (35-40)	0.9999

Leukocytes/mm^3, median (IQR)	3380 (2650-4150)	3300 (2650-4150)	3495 (2600-4530)	0.9999
Platelets ×1000/mm^3, median (IQR)	175 (152-206)	187 (172-214)	167 (142-183)	0.0276

p-values were initially calculated with Pearson´s χ^2 test for categorical variables and Mann–Whitney test for continuous variables. To account for multiple comparisons, *p*-values for categorical variables were adjusted using the Benjamini–Hochberg false discovery rate procedure, while those for continuous variables were adjusted using the Bonferroni correction. Adjusted *p*-values are reported, with values <0.05 considered statistically significant. Abbreviations: IQR, interquartile range; DWoWS, dengue without warning signs; DWWS, dengue with warning signs; SD, severe dengue.

Supplementary Tables and Figures

Table S3. Demographic characteristics, clinical manifestations and laboratory parameters of patients with detectable viremia, stratified by age.

Characteristics	<10 years	≥10 years	*p*
Demographics			
Number of patients, *n* (%)	14 (16.5)	71 (83.5)	-
Sex, *n* (%)			0.7369
Female	8 (57.1)	30 (42.3)	-
Male	6 (42.9)	41 (57.8)	-
Severity categories, *n* (%)			0.5880
DWoWS	2 (14.3)	29 (40.9)	-
DWWS	12 (85.7)	41 (57.8)	-
SD	0 (0)	1 (1.4)	-
Hospitalized, *n* (%)	4 (28.6)	22 (31.0)	0.8840
Clinical manifestations			
Constitutional, *n* (%)			
Fever	14 (100)	71 (100)	-
Headache	10 (71.4)	55 (77.5)	0.7369
Retroorbital pain	8 (57.1)	53 (74.7)	0.5880
Bilateral eyelid edema	1 (7.1)	1 (1.4)	0.5880
Conjunctival injection	5 (35.7)	30 (42.3)	0.7369
Musculoskeletal, *n* (%)			
Myalgia	12 (85.7)	65 (91.6)	0.7369
Arthralgia	9 (64.3)	58 (81.7)	0.5880
Cutaneous, *n* (%)			
Rash	6 (42.9)	23 (32.4)	0.7369
Pruritus	5 (35.7)	13 (18.3)	0.5880
Gastrointestinal, *n* (%)			
Nausea	6 (42.9)	44 (62.0)	0.5880
Vomiting	6 (42.9)	17 (23.9)	0.5880
Diarrhea	5 (35.7)	17 (23.9)	0.7369
Abdominal pain	9 (64.3)	35 (49.3)	0.7369
Hepatomegaly	0 (0)	1 (1.4)	0.7369
Splenomegaly	0 (0)	2 (2.8)	0.7369
Jaundice	0 (0)	3 (4.2)	0.7369
Oligoanuria	0 (0)	1 (1.4)	0.7369
Respiratory, *n* (%)			
Cough	3 (21.4)	14 (19.7)	0.8840
Dyspnea	1 (7.1)	2 (2.8)	0.7369
Tachypnea	1 (7.1)	1 (1.4)	0.5880
Neurological, *n* (%)			
Confusional syndrome	0 (0)	1 (1.4)	0.7369
Meningeal syndrome	0 (0)	1 (1.4)	0.7369
Hemorrhagic syndrome	1 (7.1)	4 (5.6)	0.8840
Laboratory findings			
Leukopenia, *n*/N (%)	6/10 (60.0)	37/51 (72.6)	0.7369
Thrombocytopenia, *n*/N (%)	4/9 (44.4)	9/51 (17.7)	0.5880
Hematocrit (%), median (IQR)	36 (33-39)	38 (36-40)	0.2739
Leukocytes/mm^3, median (IQR)	3730 (2980-4530)	3370 (2580-4150)	0.9999
Platelets ×1000/mm^3, median (IQR)	159 (106-210)	176 (154-206)	0.9999

Supplementary Tables and Figures

p-values were initially calculated with Pearson´s χ^2 test for categorical variables and Mann–Whitney test for continuous variables. To account for multiple comparisons, *p*-values for categorical variables were adjusted using the Benjamini–Hochberg false discovery rate procedure, while those for continuous variables were adjusted using the Bonferroni correction. Adjusted *p*-values are reported, with values <0.05 considered statistically significant. Abbreviations: IQR, interquartile range; DWoWS, dengue without warning signs; DWWS, dengue with warning signs; SD, severe dengue.

Supplementary Tables and Figures

Table S4. Demographic characteristics, clinical manifestations and laboratory parameters of patients with samples collected within 5 days after symptom onset, stratified by DENV immune status.

**Characteristics**	**Total**	**Probable Primary infection**	**Probable Secondary infection**	***p***
Demographics				
Number of patients, *n* (%)	65 (100)	34 (52.3)	31 (47.7)	-
Age group, *n* (%)				0.7856
<10 years	9 (13.9)	4 (11.8)	5 (16.1)	-
≥10 years	56 (86.2)	30 (88.2)	26 (83.9)	-
Sex, *n* (%)				0.7856
Female	29 (44.6)	14 (41.2)	15 (48.4)	-
Male	36 (55.4)	20 (58.8)	16 (51.6)	-
Disease severity, *n* (%)				0.7856
DWoWS	30 (46.2)	16 (47.1)	14 (45.2)	-
DWWS	34 (52.3)	17 (50.0)	17 (54.8)	-
SD	1 (1.5)	1 (2.9)	0 (0)	-
Hospitalized, *n* (%)	30 (46.2)	13 (38.2)	17 (54.8)	0.6048
Clinical manifestations				
Constitutional, *n* (%)				
Fever	65 (100)	34 (100)	31 (100)	-
Headache	44 (67.7)	23 (67.7)	21 (67.7)	0.9930
Retroorbital pain	48 (73.9)	28 (82.4)	20 (64.5)	0.6048
Bilateral eyelid edema	0 (0)	0 (0)	0 (0)	-
Conjunctival injection	24 (36.9)	11 (32.4)	13 (41.9)	0.6829
Musculoskeletal, *n* (%)				
Myalgia	58 (89.2)	32 (94.1)	26 (83.9)	0.6048
Arthralgia	49 (75.4)	27 (79.4)	22 (71.0)	0.6829
Cutaneous, *n* (%)				
Rash	24 (36.9)	13 (38.2)	11 (35.5)	0.9061
Pruritus	10 (15.4)	5 (14.7)	5 (16.1)	0.9076
Gastrointestinal, *n* (%)				
Nausea	38 (58.5)	19 (55.9)	19 (61.3)	0.7971
Vomiting	25 (38.5)	12 (35.3)	13 (41.9)	0.7855
Diarrhea	17 (26.2)	7 (20.6)	10 (32.3)	0.6048
Abdominal pain	26 (40.0)	14 (41.2)	12 (38.7)	0.9061
Hepatomegaly	1 (1.5)	1 (2.9)	0 (0)	0.6048
Splenomegaly	1 (1.5)	1 (2.9)	0 (0)	0.6048
Jaundice	2 (3.1)	2 (5.9)	0 (0)	0.6048
Oligoanuria	1 (1.5)	1 (2.9)	0 (0)	0.6048
Respiratory, *n* (%)				
Cough	10 (15.4)	7 (20.6)	3 (9.7)	0.6048
Dyspnea	2 (3.1)	0 (0)	2 (6.5)	0.6048
Tachypnea	1 (1.5)	0 (0)	1 (3.2)	0.6048
Neurological, *n* (%)				
Confusional syndrome	1 (1.5)	1 (2.9)	0 (0)	0.6048
Meningeal syndrome	1 (1.5)	1 (2.9)	0 (0)	0.6048
Hemorrhagic syndrome	1 (1.5)	1 (2.9)	0 (0)	0.6048
Laboratory findings				
Leukopenia, *n*/N (%)	30/47 (63.8)	16/24 (66.7)	14/23 (60.9)	0.7971
Thrombocytopenia, *n*/N (%)	14/48 (29.2)	5/24 (20.8)	9/24 (37.5)	0.6048

Supplementary Tables and Figures

Hematocrit (%), median (IQR)	38 (36-42)	37 (36-41)	38 (37-42)	0.6687
Leukocytes/mm^3, median (IQR)	3630 (2700-4890)	3565 (2715-4745)	3700 (2600-5900)	0.9999
Platelets ×1000/mm^3, median (IQR)	177 (142-206)	184 (152-207)	168 (130-204)	0.7737

p-values were initially calculated with Pearson´s χ^2 test for categorical variables and Mann–Whitney test for continuous variables. To account for multiple comparisons, *p*-values for categorical variables were adjusted using the Benjamini–Hochberg false discovery rate procedure, while those for continuous variables were adjusted using the Bonferroni correction. Adjusted *p*-values are reported, with values <0.05 considered statistically significant. Abbreviations: IQR, interquartile range; DWoWS, dengue without warning signs; DWWS, dengue with warning signs; SD, severe dengue.

Supplementary Tables and Figures

Table S5. Demographic characteristics, clinical manifestations and laboratory parameters of patients with samples collected within 5 days after symptom onset, stratified by age.

Characteristics	<10 years	≥10 years	*p*
Demographics			
Number of patients, *n* (%)	9 (13.9)	56 (86.2)	-
Sex, *n* (%)			0.0780
Female	8 (88.9)	21 (37.5)	-
Male	1 (11.1)	35 (62.5)	-
Severity categories, *n* (%)			0.0780
DWoWS	0 (0)	30 (53.6)	-
DWWS	9 (100)	25 (44.6)	-
SD	0 (0)	1 (1.8)	-
Hospitalized, *n* (%)	5 (55.6)	25 (44.6)	0.8296
Clinical manifestations			
Constitutional, *n* (%)			
Fever	9 (100)	56 (100)	-
Headache	6 (66.7)	38 (67.9)	0.9720
Retroorbital pain	6 (66.7)	42 (75.0)	0.8296
Bilateral eyelid edema	0 (0)	0 (0)	-
Conjunctival injection	3 (33.3)	21 (37.5)	0.9157
Musculoskeletal, *n* (%)			
Myalgia	8 (88.9)	50 (89.3)	0.9720
Arthralgia	6 (66.7)	43 (76.8)	0.8296
Cutaneous, *n* (%)			
Rash	2 (22.2)	22 (39.3)	0.8296
Pruritus	1 (11.1)	9 (16.7)	0.8296
Gastrointestinal, *n* (%)			
Nausea	5 (55.6)	33 (58.9)	0.9198
Vomiting	4 (44.4)	21 (37.5)	0.8296
Diarrhea	3 (33.3)	14 (25.0)	0.8296
Abdominal pain	6 (66.7)	20 (35.7)	0.5135
Hepatomegaly	0 (0)	1 (1.8)	0.8296
Splenomegaly	0 (0)	1 (1.8)	0.8296
Jaundice	0 (0)	2 (3.6)	0.8296
Oligoanuria	0 (0)	1 (1.8)	0.8296
Respiratory, *n* (%)			
Cough	1 (11.1)	9 (16.1)	0.8296
Dyspnea	0 (0)	2 (3.6)	0.8296
Tachypnea	0 (0)	1 (1.8)	0.8296
Neurological, *n* (%)			
Confusional syndrome	0 (0)	1 (1.8)	0.8296
Meningeal syndrome	0 (0)	1 (1.8)	0.8296
Hemorrhagic syndrome	0 (0)	1 (1.8)	0.8296
Laboratory findings			
Leukopenia. *n*/N (%)	5/7 (71.4)	25/40 (62.5)	0.8296
Thrombocytopenia. *n*/N (%)	5/7 (71.4)	9/41 (22.0)	0.0780
Hematocrit (%), median (IQR)	38 (35-41)	38 (37-42)	0.9999
Leukocytes/mm^3, median (IQR)	3480 (1830-4600)	3700 (2790-5455)	0.6270
Platelets ×1000/mm^3, median (IQR)	126 (18-178)	180 (151-206)	0.0903

Supplementary Tables and Figures

p-values were initially calculated with Pearson´s χ^2 test for categorical variables and Mann–Whitney test for continuous variables. To account for multiple comparisons, *p*-values for categorical variables were adjusted using the Benjamini–Hochberg false discovery rate procedure, while those for continuous variables were adjusted using the Bonferroni correction. Adjusted *p*-values are reported, with values <0.05 considered statistically significant. Abbreviations: IQR, interquartile range; DWoWS, dengue without warning signs; DWWS, dengue with warning signs; SD, severe dengue.

Supplementary Tables and Figures

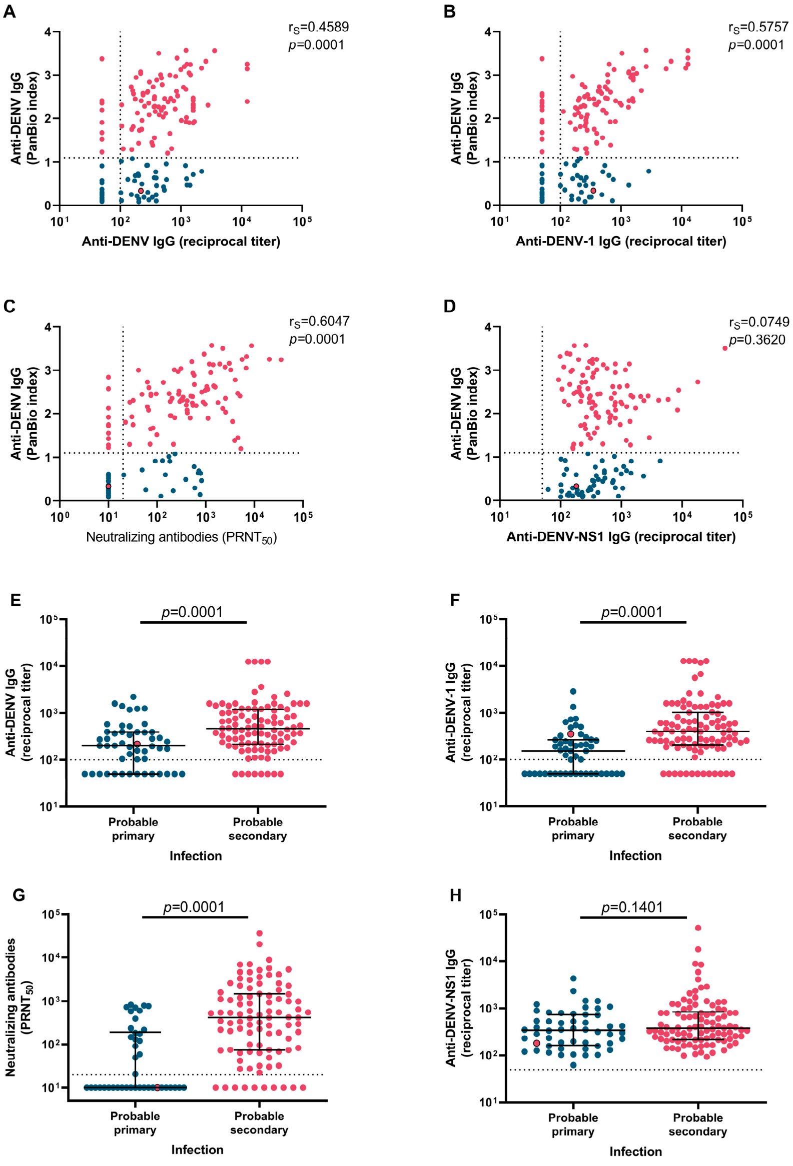

Figure S1. Correlation analyses between acute-phase dengue antibody titers and relative dengue IgG reactivity indexes. Shown are correlations between relative anti-DENV IgG PanBio indexes and (**A**) anti-DENV IgG, (**B**) anti-DENV-1 IgG, (**C**) neutralizing DENV antibodies, and (**D**) anti-DENV-NS1 IgG. Horizontal dotted lines indicate the PanBio index cut-off, and vertical dotted lines indicate in-house assay detection limits. (**E**) Anti-DENV IgG, (**F**) anti-DENV-1 IgG, (**G**) neutralizing DENV antibodies, and (**H**) anti-DENV-NS1 IgG stratified by probable primary and probable secondary infections. Median values and interquartile ranges are shown. (**A-H**) Non-reactive sera were assigned titers corresponding to half the limit of detection of the in-house assays. (**A-D**) *P*-values were initially calculated using Spearman correlation test. r_S, Spearman correlation coefficient. (**F-H**) *P*-values were initially calculated using the Mann–Whitney test. (**A-H**) All *p*-values were adjusted for multiple comparisons using the Benjamini–Hochberg false discovery rate procedure. Adjusted *p*-values are shown and values <0.05 were considered significant. Some data points may overlap in the figure, with a single dot representing more than one sample. The patient with SD is highlighted with a distinct color.

Supplementary Tables and Figures

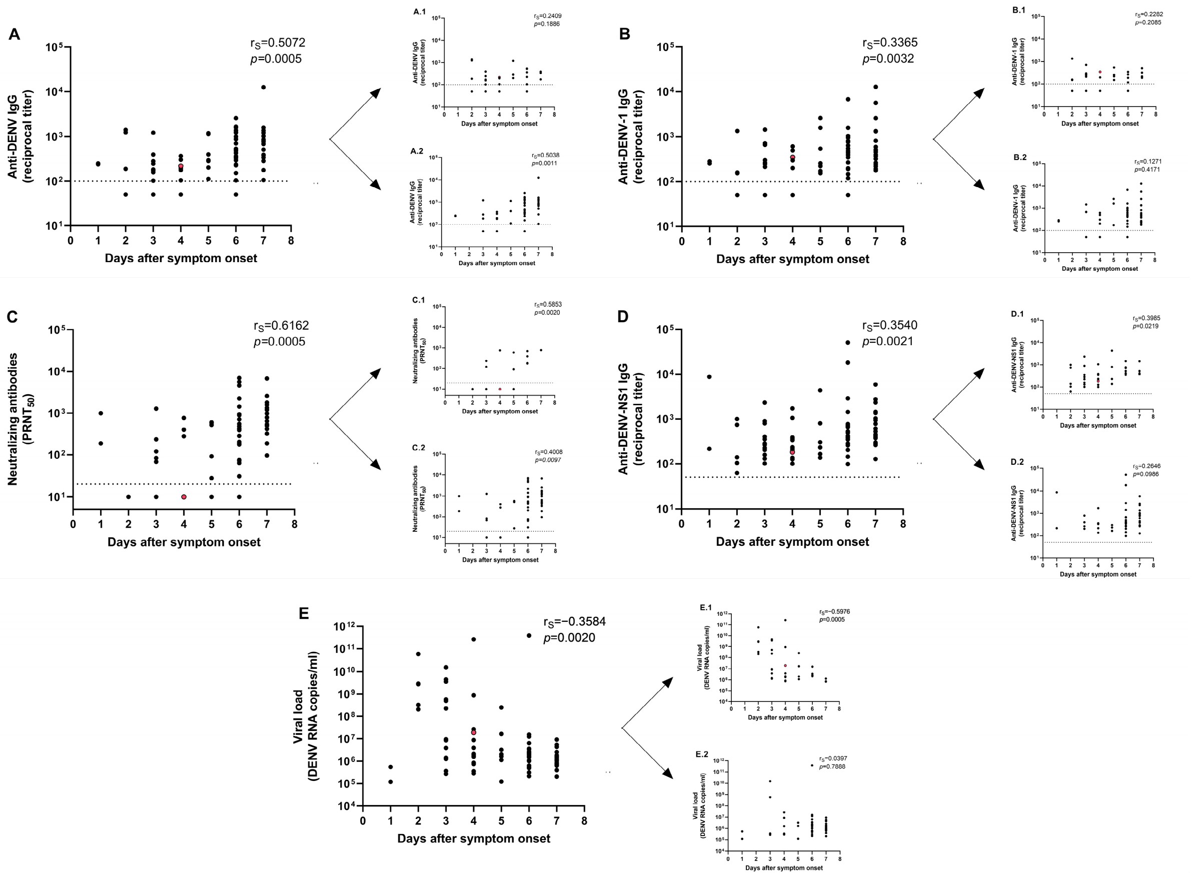

Figure S2. Correlations between timing of sample collection and acute-phase dengue antibodies or viremia in patients with detectable viremia levels. (**A**) Correlations between days after symptom onset and (**A**) anti-DENV IgG, (**B**) anti-DENV-1 IgG, (**C**) neutralizing DENV antibodies, (**D**) anti-DENV-NS1 IgG, and (**E**) DENV viremia were performed. Stratification by immune status is shown in subpanels 1 and 2: (**A.1**-**E.1**) represent probable primary infections, and (**A.2**-**E.2**) represent probable secondary infections. Horizontal dotted lines indicate assay detection limits. Non-reactive sera were assigned titers corresponding to half the limit of detection. *P* values were initially calculated with Spearman correlation test. r_S, Spearman correlation coefficient. (**A**-**E**) All *p*-values were adjusted for multiple comparisons using the Benjamini–Hochberg false discovery rate procedure. Adjusted *p*-values are shown and values <0.05 were considered significant. Some data points may overlap in the figure, with a single dot representing more than one sample. The patient with SD is highlighted in red.

Supplementary Tables and Figures

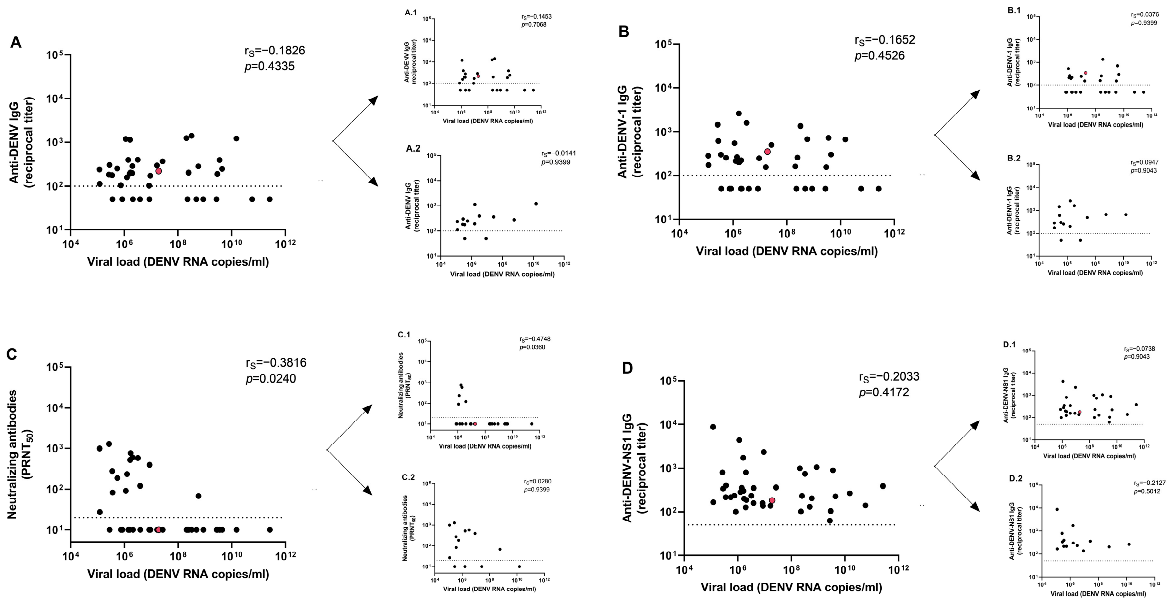

Figure S3. Correlations between acute-phase dengue antibodies and viremia in patients with detectable viremia levels and samples collected within 5 days after symptom onset. Correlations between serum viral load and (**A**) anti-DENV IgG, (**B**) anti-DENV-1 IgG, (**C**) neutralizing DENV antibodies, and (**D**) anti-DENV-NS1 IgG were performed. Stratification by immune status is shown in subpanels 1 and 2: (**A.1**-**D.1**) represent probable primary infections, and (**A.2**-**D.2**) represent probable secondary infections. Horizontal dotted lines indicate assay detection limits. Non-reactive sera were assigned titers corresponding to half the limit of detection. *P* values were initially calculated with Spearman correlation test. r_S, Spearman correlation coefficient. (**A**-**D**) All *p*-values were adjusted for multiple comparisons using the Benjamini–Hochberg false discovery rate procedure. Adjusted *p*-values are shown and values <0.05 were considered significant. Some data points may overlap in the figure, with a single dot representing more than one sample. The patient with SD is highlighted in red.

Supplementary Tables and Figures

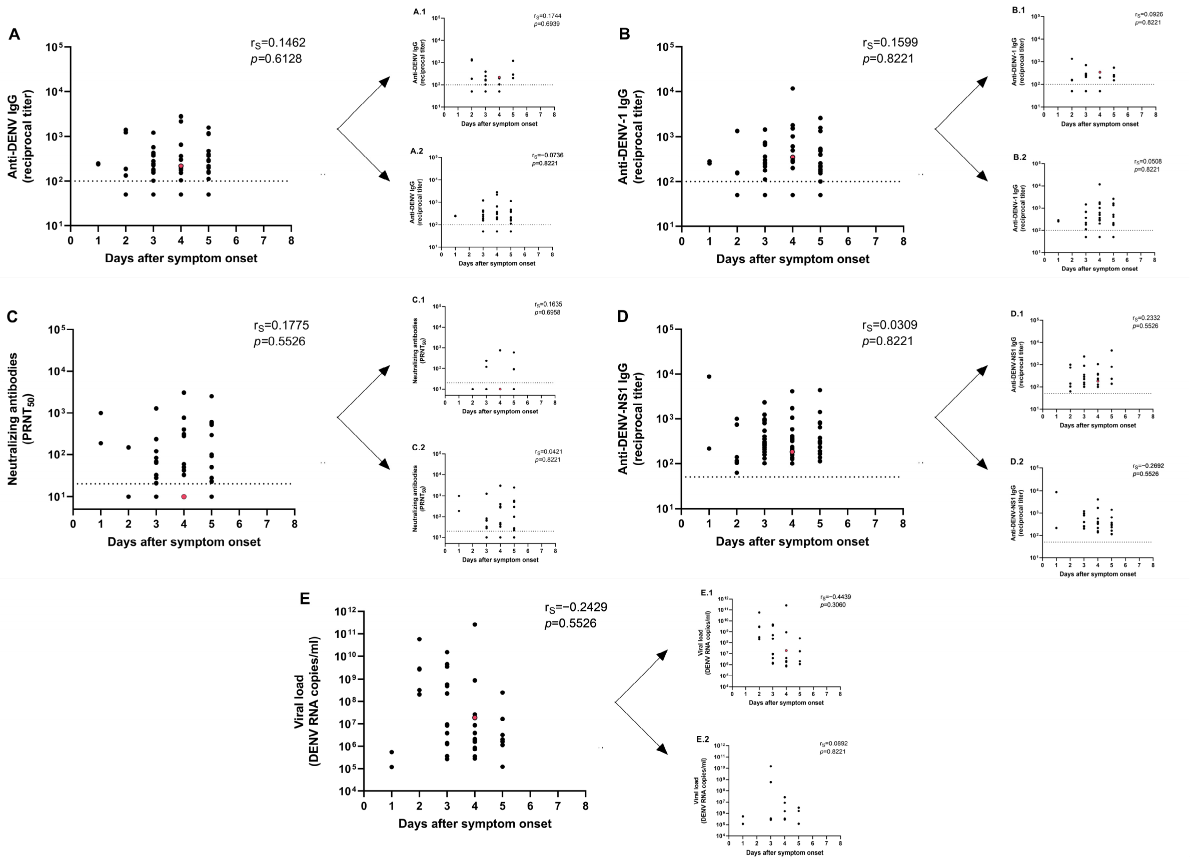

Figure S4. Correlations between timing of sample collection and acute-phase dengue antibodies or viremia in patients with detectable viremia levels and samples collected within 5 days after symptom onset. (**A**) Correlations between days after symptom onset and (**A**) anti-DENV IgG, (**B**) anti-DENV-1 IgG, (**C**) neutralizing DENV antibodies, (**D**) anti-DENV-NS1 IgG, and (**E**) DENV viremia were performed. Stratification by immune status is shown in subpanels 1 and 2: (**A.1**-**E.1**) represent probable primary infections, and (**A.2**-**E.2**) represent probable secondary infections. Horizontal dotted lines indicate assay detection limits. Non-reactive sera were assigned titers corresponding to half the limit of detection. *P* values were initially calculated with Spearman correlation test. r_S, Spearman correlation coefficient. (**A**-**E**) All *p*-values were adjusted for multiple comparisons using the Benjamini–Hochberg false discovery rate procedure. Adjusted *p*-values are shown and values <0.05 were considered significant. Some data points may overlap in the figure, with a single dot representing more than one sample. The patient with SD is highlighted in red.

Supplementary Tables and Figures

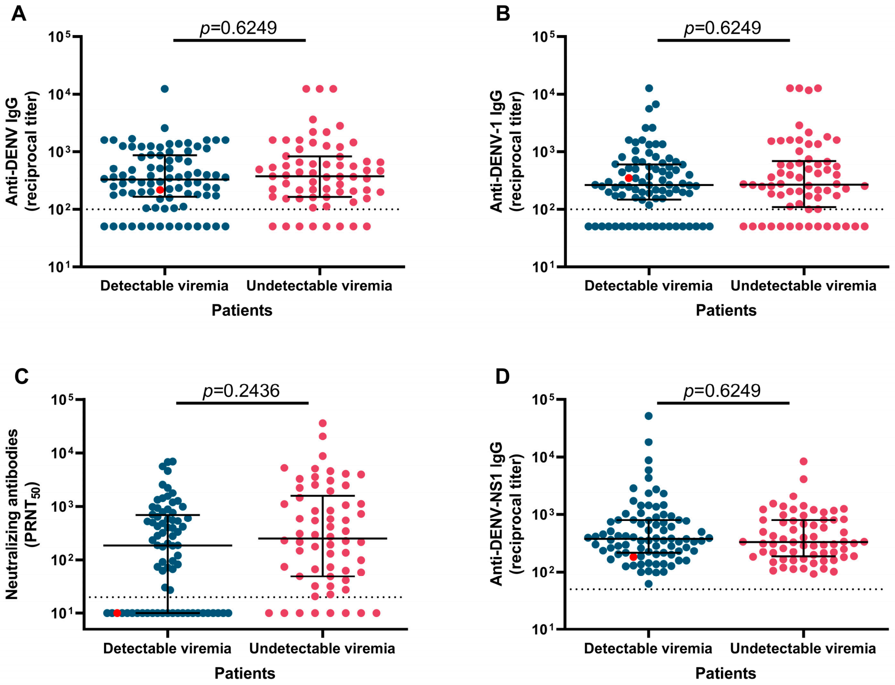

Figure S5. Acute-phase dengue antibody titers in patients with detectable or undetectable viremia. (**A**) Anti-DENV IgG, (**B**) anti-DENV-1 IgG, (**C**) neutralizing DENV antibodies, and (**D**) anti-DENV-NS1 IgG stratified by patients with detectable or undetectable viremia. Median values and interquartile ranges are shown. Non-reactive sera were assigned titers corresponding to half the limit of detection of the in-house assays. *P*-values were initially calculated using the Mann–Whitney test and adjusted for multiple comparisons using the Benjamini–Hochberg false discovery rate procedure. Adjusted *p*-values are shown and values <0.05 were considered significant. Some data points may overlap in the figure, with a single dot representing more than one sample. The patient with SD is highlighted with a distinct color.

Supplementary Tables and Figures

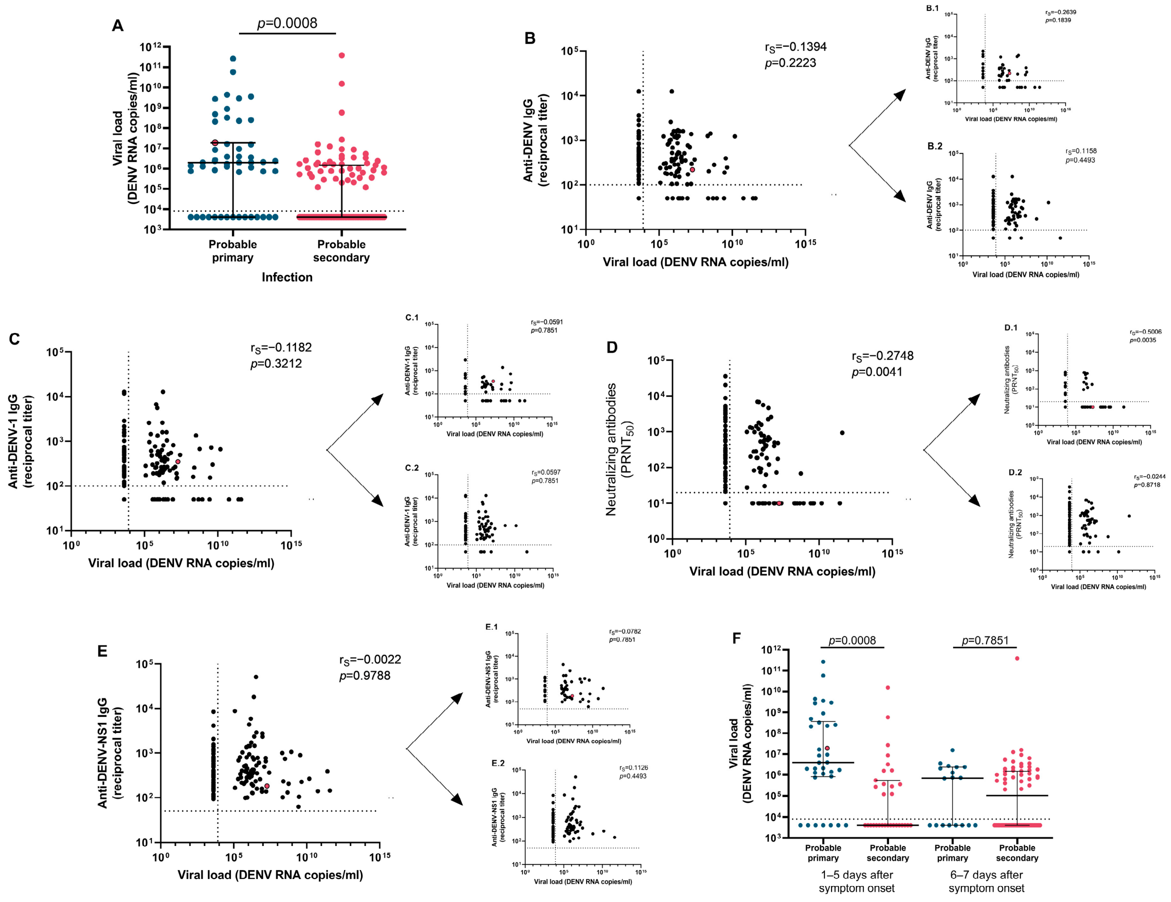

Figure S6. Correlations between acute-phase dengue antibodies and viremia in the overall study population. (**A**) Serum viral load was analyzed according to DENV immune status. Non-detectable viremia samples were assigned viremia levels corresponding to half the lower limit of detection. Correlations between serum viral load and (**B**) anti-DENV IgG, (**C**) anti-DENV-1 IgG, (**D**) neutralizing DENV antibodies, and (**E**) anti-DENV-NS1 IgG were performed. Stratification by immune status is shown in subpanels 1 and 2: (**B.1**-**E.1**) represent probable primary infections, and (**B.2**-**E.2**) represent probable secondary infections. (**F**) Serum viral load was analyzed according to DENV immune status and stratified by days after symptom onset. (**A**, **F**) Median and interquartile ranges are shown. *P* values were initially calculated with Mann-Whitney test. For (**A**, **F**) horizontal dotted lines indicate RT-qPCR detection limits. For (**B**-**E**) horizontal dotted lines indicate antibody assay detection limits. Non-reactive sera were assigned titers corresponding to half the limit of detection. *P* values were initially calculated with Spearman correlation test. r_S, Spearman correlation coefficient. (**A**-**F**) All *p*-values were adjusted for multiple comparisons using the Benjamini–Hochberg false discovery rate procedure. Adjusted *p*-values are shown and values <0.05 were considered significant. Some data points may overlap in the figure, with a single dot representing more than one sample. The patient with SD is highlighted in red.

Supplementary Tables and Figures

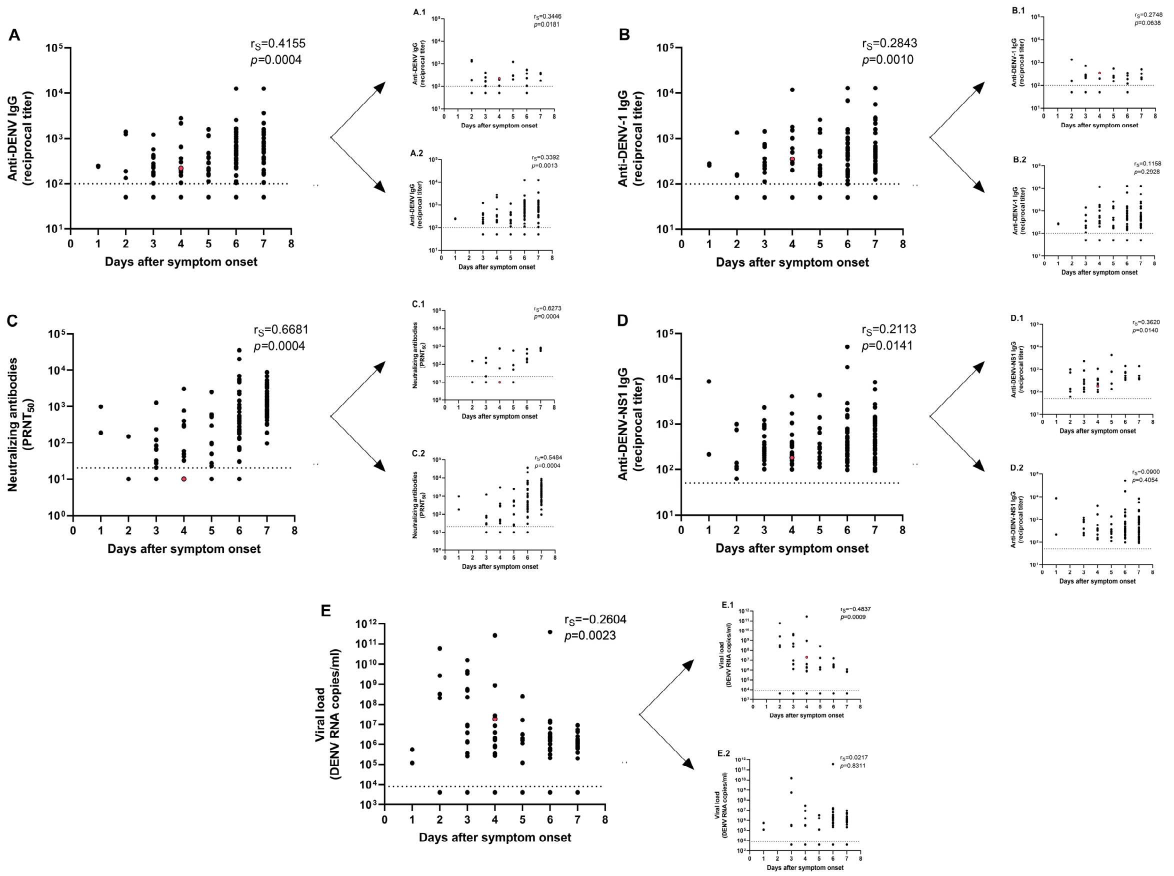

Figure S7. Correlations between timing of sample collection and acute-phase dengue antibodies or viremia in the overall study population. Non-detectable viremia samples were assigned viremia levels corresponding to half the lower limit of detection. Correlations between days after symptom onset and (**A**) anti-DENV IgG, (**B**) anti-DENV-1 IgG, (**C**) neutralizing DENV antibodies, (**D**) anti-DENV-NS1 IgG, and (**E**) DENV viremia were performed. Stratification by immune status is shown in subpanels 1 and 2: (**A.1**-**E.1**) represent probable primary infections, and (**A.2**-**E.2**) represent probable secondary infections. Horizontal dotted lines indicate assay detection limits. Non-reactive sera were assigned titers corresponding to half the limit of detection. *P* values were initially calculated with Spearman correlation test. r_S, Spearman correlation coefficient. (**A**-**E**) All *p*-values were adjusted for multiple comparisons using the Benjamini–Hochberg false discovery rate procedure. Adjusted *p*-values are shown and values <0.05 were considered significant. Some data points may overlap in the figure, with a single dot representing more than one sample. The patient with SD is highlighted in red.

Supplementary Tables and Figures

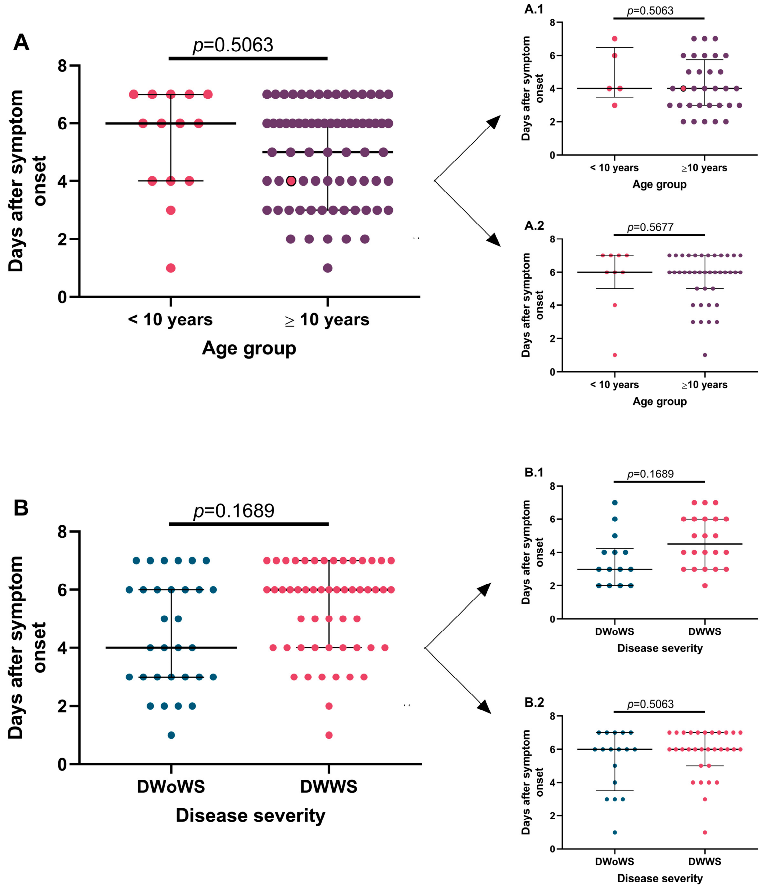

Figure S8. Timing of sample collection by age group and disease severity in patients with detectable viremia levels. Days after symptom onset were analyzed across (**A**) age groups, and (**B**) disease severity categories. Stratification by immune status is shown in subpanels 1 and 2: (**A.1**, **B.1**) represent probable primary infections, and (**A.2**, **B.2**) represent probable secondary infections. Median and interquartile ranges are shown. *P*-values were initially calculated with Mann–Whitney test. All *p*-values were adjusted for multiple comparisons using the Benjamini–Hochberg false discovery rate procedure. Adjusted *p*-values are shown and values <0.05 were considered significant. Some data points may overlap in the figure, with a single dot representing more than one sample. The patient with SD is highlighted with a distinct color. DWoWS, dengue without warning signs; DWWS, dengue with warning signs.

Supplementary Tables and Figures

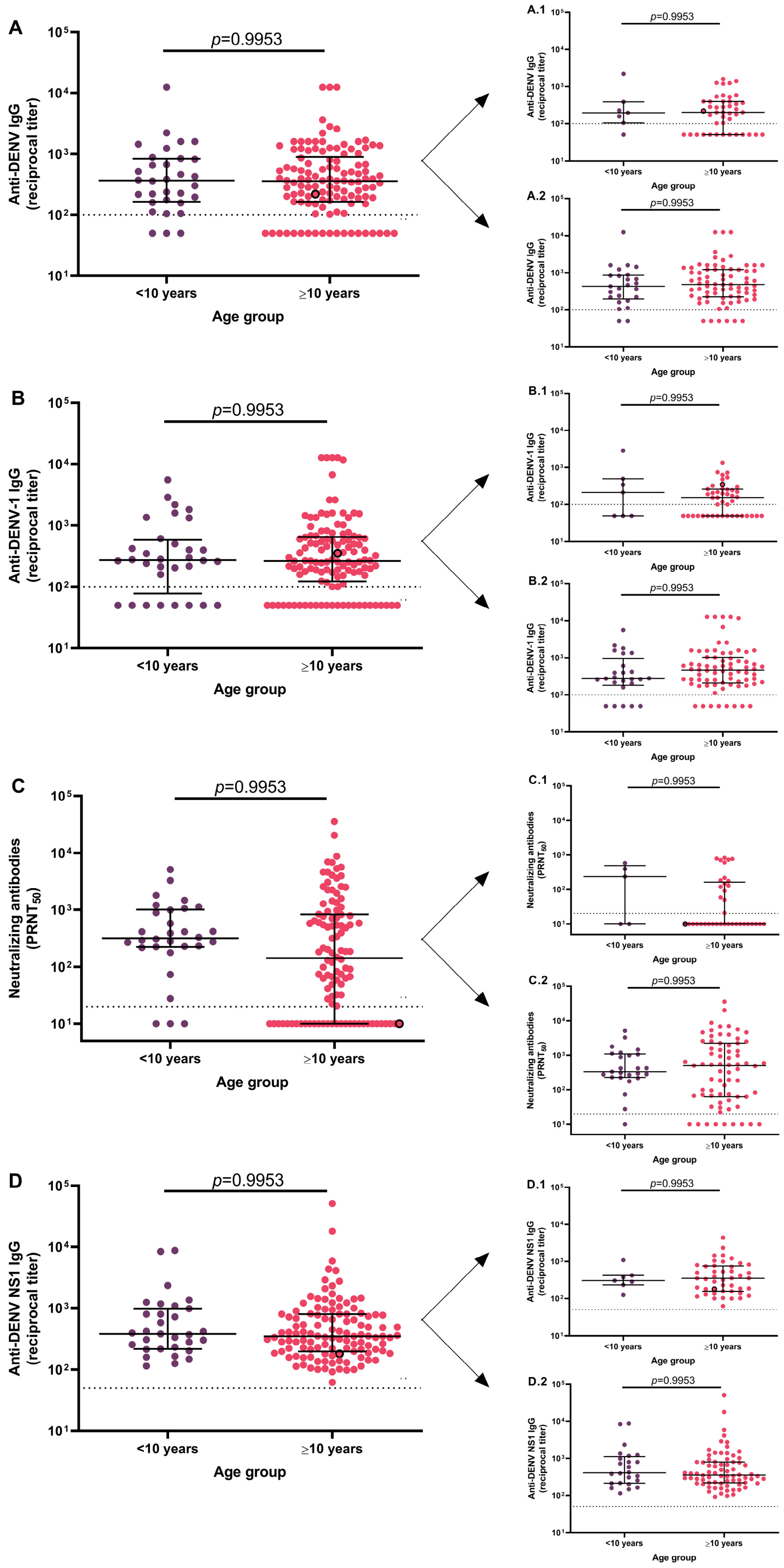

Figure S9. Acute-phase dengue antibodies and patient age. (**A**) Anti-DENV IgG, (**B**) anti-DENV-1 IgG, (**C**) neutralizing DENV antibodies, and (**D**) anti-DENV-NS1 IgG were analyzed in relation to patient age. Stratification by immune status is shown in subpanels 1 and 2: (**A.1**-**D.1**) represent probable primary infections, and (**A.2**-**D.2**) represent probable secondary infections. Median and interquartile ranges are shown. *P*-values were initially calculated with Mann–Whitney test. All *p*-values were adjusted for multiple comparisons using the Benjamini–

Supplementary Tables and Figures

Hochberg false discovery rate procedure. Adjusted *p*-values are shown and values <0.05 were considered significant. Horizontal dotted lines indicate assay detection limits. Non-reactive sera were assigned titers corresponding to half the limit of detection. Some data points may overlap in the figure, with a single dot representing more than one sample. The patient with SD is highlighted with a distinct color.

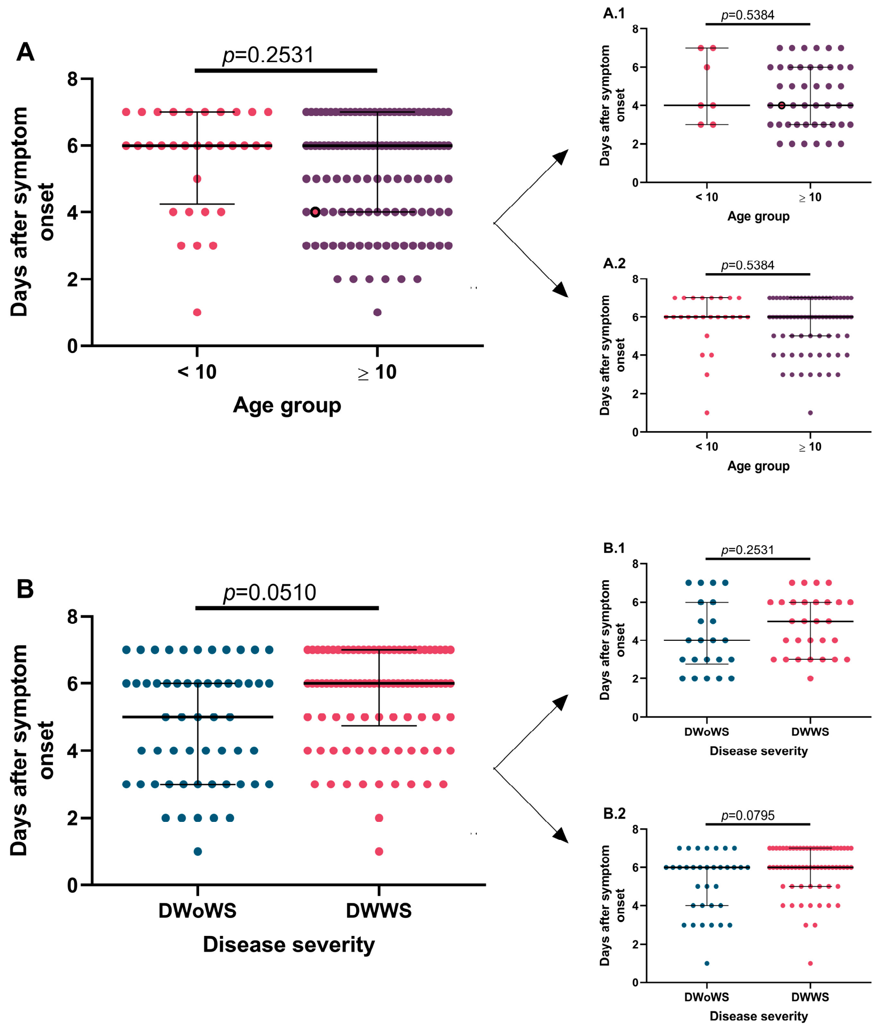

Figure S10. Timing of sample collection by age group and disease severity in the overall study population. Days after symptom onset were analyzed across (**A**) age groups, and (**B**) disease severity categories. Stratification by immune status is shown in subpanels 1 and 2: (**A.1**, **B.1**) represent probable primary infections, and (**A.2**, **B.2**) represent probable secondary infections. Median and interquartile ranges are shown. *P*-values were initially calculated with Mann–Whitney test. All *p*-values were adjusted for multiple comparisons using the Benjamini–Hochberg false discovery rate procedure. Adjusted *p*-values are shown and values <0.05 were considered significant. Some data points may overlap in the figure, with a single dot representing more than one sample. The patient with SD is highlighted with a distinct color. DWoWS, dengue without warning signs; DWWS, dengue with warning signs.

Supplementary Tables and Figures

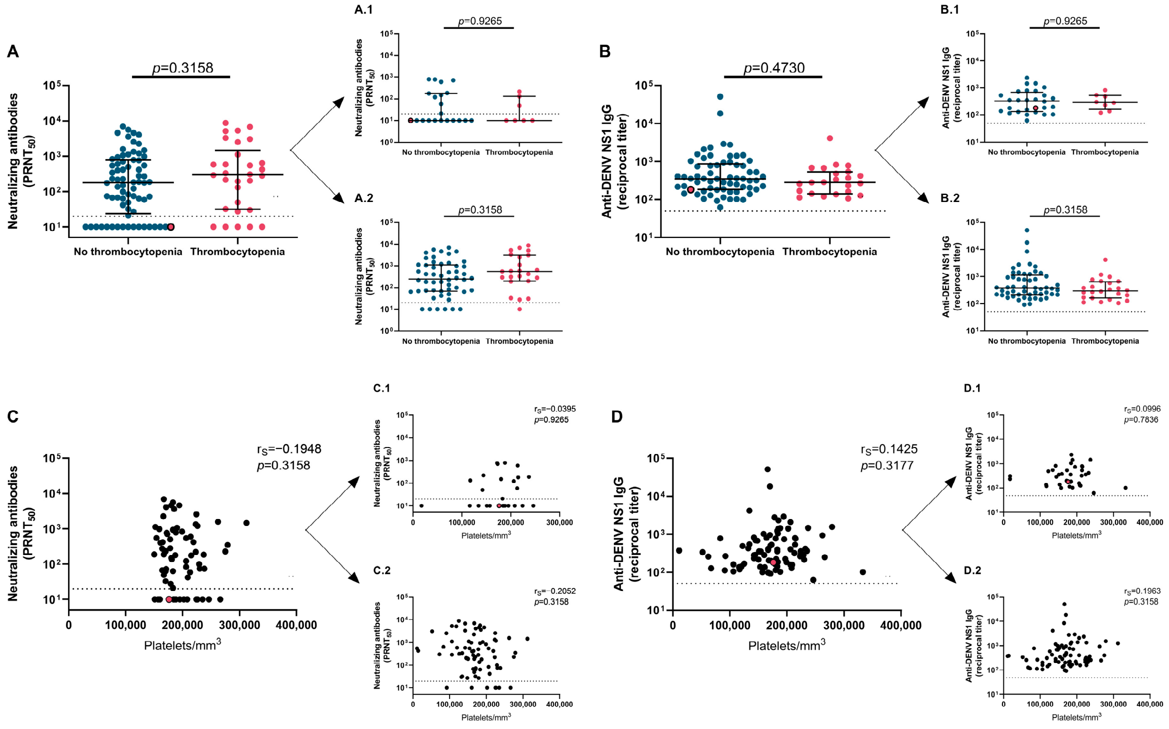

Figure S11. Association between acute-phase dengue antibodies and thrombocytopenia in adolescents. (**A**) Neutralizing DENV-1 antibodies and (**B**) anti-DENV-NS1 IgG were compared in patients with or without thrombocytopenia. Correlations between platelet counts and (**C**) neutralizing DENV-1 antibodies and (**D**) anti-DENV-NS1 IgG were performed. Stratification by immune status is shown in subpanels 1 and 2: (**A.1-D.1**) represent probable primary infections, and (**A.2-D.2**) represent probable secondary infections. (**A**, **B**) Median and interquartile ranges are shown. *P* values were initially calculated with Mann–Whitney test. For (**C**, **D**) *p* values were initially calculated with Spearman correlation test. r_S, Spearman correlation coefficient. (**A-D**) All *p*-values were adjusted for multiple comparisons using the Benjamini–Hochberg false discovery rate procedure. Adjusted *p*-values are shown and values <0.05 were considered significant. Horizontal dotted lines indicate assay detection limits. Non-reactive sera were assigned titers corresponding to half the limit of detection. Some data points may overlap in the figure, with a single dot representing more than one sample. The patient with SD is highlighted with a distinct color.

Supplementary Tables and Figures

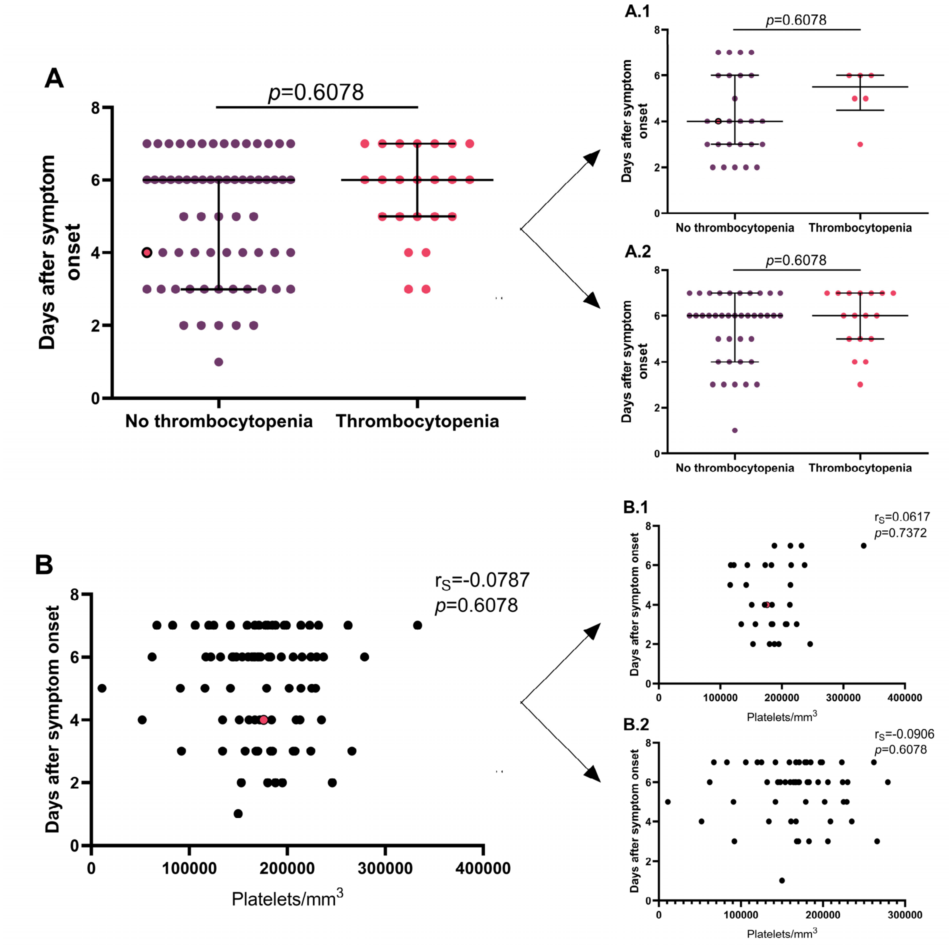

Figure S12. Timing of sample collection and thrombocytopenia in adolescents. (**A**) Days after symptom onset were analyzed in adolescents with or without thrombocytopenia. (**B**) Correlation between days after symptom onset and platelet counts. Stratification by immune status is shown in subpanels 1 and 2: (**A.1**, **B.1**) represent probable primary infections, and (**A.2**, **B.2**) represent probable secondary infections. (**A**) Median and interquartile ranges are shown. *P*-values were initially calculated with Mann-Whitney test. (**B**) *P*-values were initially calculated with Spearman correlation test. r_S, Spearman correlation coefficient. (**A**, **B**) All *p*-values were adjusted for multiple comparisons using the Benjamini–Hochberg false discovery rate procedure. Adjusted *p*-values are shown and values <0.05 were considered significant. Some data points may overlap in the figure, with a single dot representing more than one sample. The patient with SD is highlighted with a distinct color.
